# Regulation of Cell Wall-Bound Invertase in Pepper Leaves by *Xanthomonas campestris* pv. *vesicatoria* Type Three Effectors

**DOI:** 10.1371/journal.pone.0051763

**Published:** 2012-12-14

**Authors:** Sophia Sonnewald, Johannes P. R. Priller, Julia Schuster, Eric Glickmann, Mohammed-Reza Hajirezaei, Stefan Siebig, Mary Beth Mudgett, Uwe Sonnewald

**Affiliations:** 1 Lehrstuhl für Biochemie, Friedrich-Alexander Universität Erlangen-Nürnberg, Erlangen, Germany; 2 Institut für Pflanzengenetik und Kulturpflanzenforschung, Gatersleben, Germany; 3 Department of Biology, Stanford University, Stanford, California, United States of America; Umeå Plant Science Centre, Sweden

## Abstract

*Xanthomonas campestris* pv. *vesicatoria (Xcv)* possess a type 3 secretion system (T3SS) to deliver effector proteins into its *Solanaceous* host plants. These proteins are involved in suppression of plant defense and in reprogramming of plant metabolism to favour bacterial propagation. There is increasing evidence that hexoses contribute to defense responses. They act as substrates for metabolic processes and as metabolic semaphores to regulate gene expression. Especially an increase in the apoplastic hexose-to-sucrose ratio has been suggested to strengthen plant defense. This shift is brought about by the activity of cell wall-bound invertase (cw-Inv). We examined the possibility that *Xcv* may employ type 3 effector (T3E) proteins to suppress cw-Inv activity during infection. Indeed, pepper leaves infected with a T3SS-deficient *Xcv* strain showed a higher level of cw-Inv mRNA and enzyme activity relative to *Xcv* wild type infected leaves. Higher cw-Inv activity was paralleled by an increase in hexoses and mRNA abundance for the *pathogenesis-related* gene *PRQ.* These results suggest that *Xcv* suppresses cw-Inv activity in a T3SS-dependent manner, most likely to prevent sugar-mediated defense signals. To identify *Xcv* T3Es that regulate cw-Inv activity, a screen was performed with eighteen *Xcv* strains, each deficient in an individual T3E. Seven *Xcv* T3E deletion strains caused a significant change in cw-Inv activity compared to *Xcv* wild type. Among them, *Xcv* lacking the *xopB* gene (*Xcv* Δ*xopB*) caused the most prominent increase in cw-Inv activity. Deletion of *xopB* increased the mRNA abundance of *PRQ* in *Xcv* Δ*xopB-*infected pepper leaves, but not of *Pti5* and *Acre31,* two PAMP-triggered immunity markers. Inducible expression of XopB in transgenic tobacco inhibited *Xcv*-mediated induction of cw-Inv activity observed in wild type plants and resulted in severe developmental phenotypes. Together, these data suggest that XopB interferes with cw-Inv activity *in planta* to suppress sugar-enhanced defense responses during *Xcv* infection.

## Introduction

*Xanthomonas campestris* pv. *vesicatoria (Xcv*, recently re-classified as *X. euvesicatoria)* is a Gram-negative bacterium that causes bacterial spot disease in tomato (*Solanum lycopersicum*) and pepper (*Capsicum annuum*) plants. The bacteria enter the plant through small wounds or stomata and colonize the intercellular space (apoplast). Infection of susceptible plants induces macroscopically visible disease symptoms, so-called water-soaked lesions that become later necrotic [1]. The pathogenicity of *Xcv* and many other pathogenic bacteria depends on a type 3 secretion system (T3SS) which mediates the secretion of bacterial effector proteins, termed type 3 effector proteins (T3Es), directly into the host cell [2]. The T3SS machinery is encoded by the *hrp* (*h*ypersensitive *r*esponse and *p*athogenicity) gene cluster [3]. Mutants lacking a functional T3SS cannot translocate effector proteins and are unable to elicit a defense-associated hypersensitive response in resistant plants or to establish disease in susceptible plants [4].

*Xcv* is predicted to deliver at least 28 T3Es into plant cells. Candidate T3Es were shown to be secreted experimentally and/or were predicted based on bioinformatic analyses of the complete genomic sequence [5]–[7]. In a compatible interaction, these effector proteins are important virulence factors that modify cellular processes to suppress host immune responses and to provide a favorable environment for bacterial propagation [8], [9]. In an incompatible interaction, plant resistance (R) proteins recognize the action of certain T3Es in resistant host plants. This recognition is often associated with a hypersensitive response (HR), a rapid, localized cell death response preventing bacterial multiplication [4]. Thus, T3E proteins can either act as virulence and/or avirulence factors that promote bacterial growth or induce plant immunity.

Plant immunity relies on two overlapping signaling pathways that perceive potential pathogens and trigger appropriate defense responses [10], [11]. The first, referred to as PAMP-triggered immunity (PTI), is induced by the recognition of conserved pathogen- (microbe-) associated molecular pattern (P/MAMP) such as flagellin or lipopolysaccharides by specific extracellular receptors termed pattern recognition receptors [11], [12]. This perception elicits a variety of downstream responses like the production of reactive oxygen species, a calcium burst, the stimulation of mitogen-activated protein kinase (MAPK) cascades, an increased expression of numerous pathogenesis-related (PR) proteins, as well as cell wall strengthening by the deposition of the glucose polymer callose (recently summarized by [12]). The second layer of defense is referred to as effector-triggered immunity (ETI). During ETI, R-proteins directly or indirectly recognize specific T3Es and initiate defense signal transduction cascades similar to that for PTI [10], [11]. In general, ETI responses are stronger and more prolonged compared to PTI responses.

Activation of plant defense responses, like reinforcement of the cell wall, production of reactive oxygen species or the accumulation of antimicrobial compounds requires energy, reducing power and carbon skeletons that can be fueled by carbohydrates [13]–[15]. In fact, the availability of sugars was shown to play a role for plant resistance against pathogens and the phenomenon of high-sugar resistance was recognized in the mid-1950’s [16]. The increased metabolic activity is probably fed by an enhanced flow of sucrose to the site of infection [17] or reduced sucrose export [18]. This is accompanied by increased expression and activity of cell wall-bound invertase (cw-Inv), which has been observed upon infection with plant pathogens in numerous studies [13], [19]–[22]. Cw-Inv catalyzes the cleavage of the transport sugar sucrose into glucose and fructose, which are not transported in the phloem and therefore accumulate at the site of formation. Generally, cw-Inv is mainly active in carbohydrate consuming *sink* tissue, while its activity is usually low in mature *source* leaves with high photosynthetic capacity [17], [21]. Therefore, cw-Inv is thought to be a key enzyme for supplying sink organs with carbohydrates and was shown to be important for developmental processes like seed or pollen development (summarized in [23]). Moreover, expression of cw-Inv was shown to be stimulated by glucose, sucrose, phytohormones and by treatment with different elicitors like chitosan and polygalacturonic acid and with non-metabolizable sugars [24]–[27]. Hence this enzyme appears to play an important role to various abiotic and biotic stress responses.

An accumulation of soluble sugars has been observed along with an increased cw-Inv activity in response to pathogen attack in both compatible [20], [21], [22] and incompatible interactions [13], [22], but this occurs more rapidly and to greater extent in incompatible interactions. Hence, a fast increase of hexoses appeared to be important for an efficient and successful defense response. This is supported by the observation that high levels of sugars by expressing a yeast invertase in either the apoplast or in the vacuole of transgenic tobacco plants mediated resistance against potato virus Y [14], [20]. In contrast, repression of cw-Inv activity in tobacco using an RNAi approach resulted in a lower amount of available hexoses and caused an impaired defense response following infection with *Phytophthora nicotianae* [13]. Increasing sugar content during later infection stages, however are likely to support pathogen nutrition resulting in disease development [28]. Interestingly, infection of cw-Inv-silenced tomato plants with *Xcv* provided no evidence that reduced hexose levels restricted bacterial growth [21]. Instead, lower cw-Inv activity was linked to delayed symptom development, a slower reduction of photosynthesis and a decelerated rate of pathogen-induced senescence which was most likely caused by the absence of hexose signals [21].

In fact, besides being a source of carbon skeletons and energy, soluble sugars are known to act as signaling molecules. For instance, increasing glucose levels lead to a repression of genes involved in photosynthesis and reserve mobilization and to an up-regulation of defense gene expression (summarized in [29]). Accordingly, down-regulation of photosynthetic gene expression and activity together with an induced expression of *PR* genes has been found in numerous plant-pathogen interactions (for review see [30], [31]). Thus in a compatible interaction, the induction of cw-Inv activity may also contribute to the regulation of defense responses and photosynthesis by the generation of hexose signals.

Understanding the molecular function, mechanisms and structure of bacterial T3Es as well as the identification of their host targets is a major goal to unravel the molecular basis of plant-bacterial interactions. T3Es mimic eukaryotic proteins in structure and function and use a variety of biochemical mechanisms to target specific host proteins [32], [33]. However, the functional analysis of T3Es is often hindered by their overlapping properties and by the fact that inactivation of individual effectors often has no significant effect on bacterial virulence [34], [35]. Nevertheless, for some *Xcv* T3Es, the molecular function and first host target proteins could be identified [36], [37]. For instance, AvrBs2, which is present in many *Xanthomonas* pathovars, was shown to be important for their virulence [38]. Recently, Zhao et al. [39] demonstrated that AvrBs2 contains an active glycerolphosphodiesterase domain which is required for its virulence function but not for its recognition by the Bs2 resistance protein. AvrBs3, another T3E, is the best characterized member of a large family of transcription activator like T3Es [36], [40]. They harbor functional domains typical for eukaryotic transcription activators, like a nuclear localization signal, an acid activation domain and a central repeat region consisting of nearly identical repeats of usually 34 amino acids which mediate host DNA binding in a highly specific manner. AvrBs3 was shown to bind directly to the promoter region of target genes like *upa20* (up-regulated by AvrBs3) which encodes a basic helix-loop-helix transcription factor and is a cell-growth regulator causing the enlargement of mesophyll cells observed in susceptible pepper plants [40]. XopN interacts with an atypical receptor-like kinase and 14-3-3 proteins in tomato and suppresses PTI during an early stage of infection [41], [42]. XopJ and XopX, two other T3Es from *Xcv* were shown to suppress basal defense by either inhibiting secretion or an yet unknown mechanism, respectively [43], [44]. Moreover, several effector proteins of *Xcv* inhibited growth of yeast cells under normal or stress conditions and also induced cell death or chlorosis when transiently expressed in tomato or *Nicotiana benthamiana* [45].

Here we investigated the role of cw-Inv during the compatible interaction between *Xcv* and its host pepper. We examined the possibility that *Xcv* employs T3Es to modulate cw-Inv activity during infection to promote pathogenesis. We show that induction of cw-Inv is suppressed by the translocation of T3Es, which may prevent the generation of hexose-mediated signals. Eighteen different *Xcv* mutants deficient in individual T3Es were tested for their effect on cw-Inv activity upon infection of pepper leaves. Among them, infection with *Xcv* Δ*xopB* caused the strongest and most robust induction of cw-Inv activity similar to a T3SS-deficient mutant (*Xcv* Δ*hrpB1*), indicating that this effector plays a key role in suppression of cw-Inv activity. Moreover, inducible expression of XopB in transgenic tobacco inhibited *Xcv*-mediated induction of cw-Inv activity observed in wild type plants and resulted in severe developmental phenotypes. These data suggest that XopB interferes with cw-Inv activity *in planta* to suppress sugar-enhanced defense responses during *Xcv* infection.

## Results

### *Xcv* Suppresses cw-Inv Expression and Activity of Pepper Leaves in a T3SS-dependent Manner

An increase in *cw-Inv* mRNA expression has been observed after infection of plants with both virulent and avirulent pathogens, however the timing of induction was different [30], [31]. An early induction of cw-Inv transcripts and the accompanied accumulation of hexoses at the infection site are thought to meet the increased energy demand for defense responses and have been associated with plant resistance during incompatible interactions [15], [28]. However, the role of cw-Inv during compatible interactions is less clear. Here we tested the hypothesis that *Xcv* T3Es contribute to successful colonization of pepper plants by suppressing cw-Inv activity to prevent the generation of hexose signals and thereby suppress the down-regulation of photosynthesis and the activation of defense responses.

**Figure 1:**
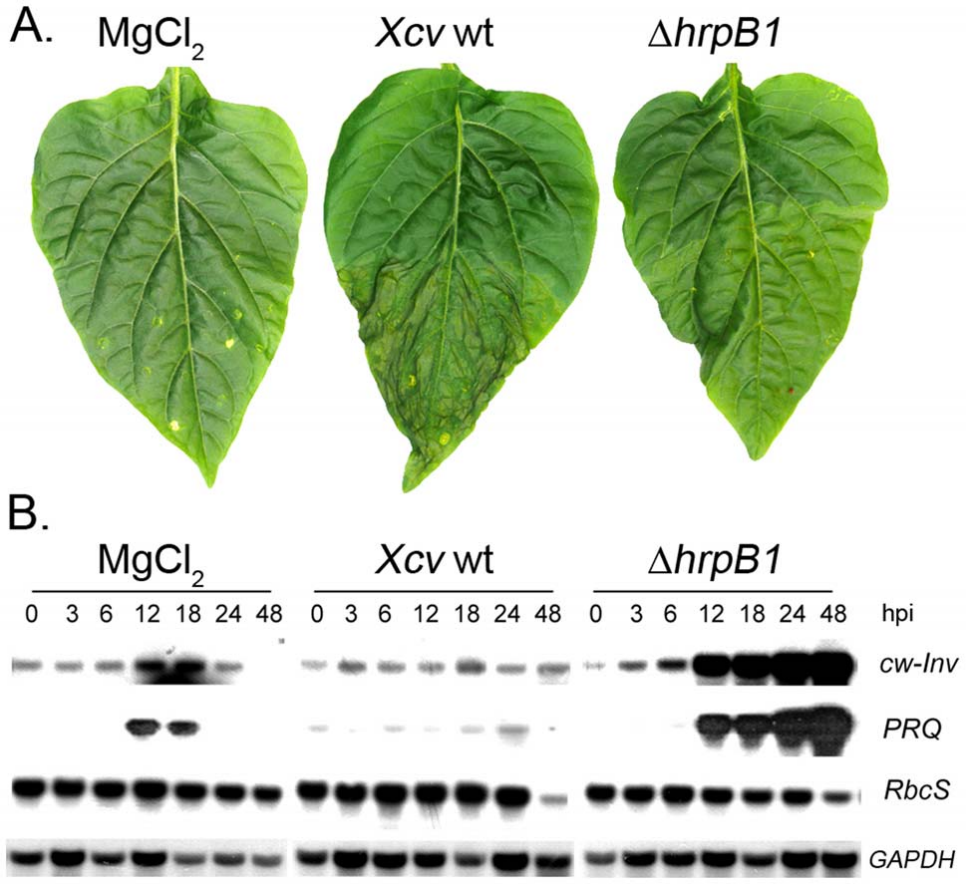
Infection of susceptible pepper leaves with *Xcv* wild type or with the T3SS-deficient *Xcv* Δ*hrpB1* strain. Impact on symptom development and transcript accumulation of *cw-Inv, PRQ* and *RbcS*. Fully mature leaves of young pepper plants were infected with the *Xcv* wild type (wt), the *Xcv* Δ*hrpB1* using a concentration of 5×10^8^ cfu ml^−1^, and as control with 10 mM MgCl_2_. A.) Formation of disease symptoms after *Xcv* wild type inoculation of susceptible pepper leaves. Only the lower halves of leaves were infiltrated. Pictures were taken 3 days post infection. B.) For Northern Blot analysis total RNA was isolated from leaf material taken before (0 h) and 3, 6, 12, 18, 24 and 48 hours post infection (hpi). Thirty micrograms of total RNA were loaded per each lane. The Northern blot was probed with [^32^]P-labeled cDNA fragments of *cw-Inv*, *PRQ*, *RbcS* and cytosolic *GAPDH* as control. Results of a representative experiment are shown which have been repeated three times.

Susceptible pepper plants were infected with *Xcv* wild type strain 85-10 and the T3SS- deficient mutant strain *Xcv* Δ*hrpB1* [46], which cannot deliver T3Es into plant cells, and the response on cw-Inv mRNA expression and enzyme activity was tested. These experiments were carried out in a contained growth cabinet (see Materials and Methods) and the bacterial density used for infection was 5×10^8^ colony forming units (cfu) ml^−1^. Under these conditions, the first disease symptoms appeared 2 days after *Xcv* wild type infection, which were visible as water-soaked lesions (Fig. 1A). Later, the *Xcv*-infected leaves became chlorotic and necrotic and eventually abscised. In contrast, infection with *Xcv* Δ*hrpB1* caused no visible symptoms at the same time point (Fig. 1A). For control purposes, pepper leaves were infiltrated with 10 mM MgCl_2_ (Mock-control).

To investigate the impact of *Xcv* infection on *cw-Inv* expression, total RNA was isolated from infected pepper leaves and subjected to a Northern blot analysis. In addition, we also monitored the mRNA expression of the *pathogenesis-related protein Q* (*PRQ*) and *ribulose-1,5-bisphosphate carboxylase (RbcS)*. The *PRQ* gene is induced upon infection and sugar floating [47], and was used as a marker gene for sugar-mediated defense. The *RbcS* gene served as a marker for photosynthetic gene expression. As shown in Fig. 1B, a low level of *cw-Inv* and *PRQ* transcripts was detected at 12 h and 18 h after MgCl_2_ infiltration in control leaves, reflecting the wounding response in the host leaves due to the infiltration process. Infection with *Xcv* wild type caused no significant changes in mRNA abundance for *cw-Inv* or *PRQ*. The mRNA level for *RbcS* however was reduced in *Xcv-*infected leaves at 48 h post-inoculation compared to the MgCl_2_ inoculated leaves (Fig. 1B). Reduced *RbcS* mRNA levels correlated with the appearance of disease symptom development at 48 h post-inoculation (Fig. 1A and B). In contrast, infection with *Xcv* Δ*hrpB1* resulted in a progressive accumulation of *cw-Inv*- and *PRQ*-specific transcripts from 12 to 48 h post-inoculation, whereas *RbcS* mRNA levels were reduced at 48 h post-inoculation (Fig. 1B). These data suggest that one or more T3Es from *Xcv* may regulate the expression and/or stability of *cw-Inv* in pepper leaves during infection.

**Figure 2:**
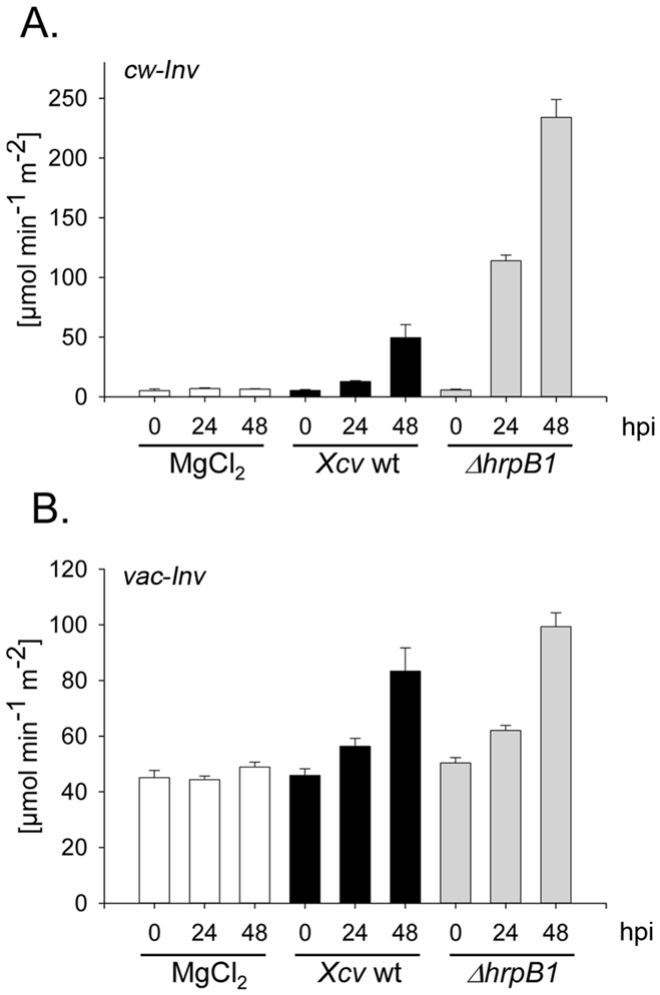
Activities of cell wall-bound and vaculoar invertase in susceptible pepper leaves following infection with *Xcv* wild type or with the TTSS-deficient *Xcv* Δ*hrpB1* strain. Leaves of susceptible pepper plants were infected with the *Xcv* wild type (wt), the *Xcv* Δ*hrpB1* using a concentration of 5×10^8^ cfu ml^−1^, and as control with 10 mM MgCl_2_. Activities of cell wall-bound acid invertase (cw-Inv) (A.) and acid soluble, vacuolar invertase (vac-Inv) (B.) were measured from source leaves before (0 h), 24 and 48 hours post infection (hpi) with *Xcv* wild type (wt) (black bars), *Xcv* Δ*hrpB1* (grey bars*)* or 10 mM MgCl_2_ (white bars). Each value represents the mean ± SE of six samples taken from three different plants. The experiment was repeated three times with similar results.

Next, cw-Inv activity was determined following infection with *Xcv* wild type or *Xcv* Δ*hrpB1* to analyze whether changes in mRNA abundance were also reflected at the enzyme activity level. *Xcv* wild type infection led to a stimulation of cw-Inv enzymatic activity 48 h after infiltration (Fig. 2A), which is in accordance with previous studies [21], [31]. However, a rapid and stronger induction of cw-Inv activity was measured in pepper leaves infected with the T3SS-deficient strain *Xcv* Δ*hrpB1* (Fig. 2A), coinciding with changes in *cw-Inv* mRNA abundance (Fig. 1B). Control pepper leaves inoculated with 10 mM MgCl_2_ showed no changes in cw-Inv activity at 24 or 48 h post-inoculation (Fig. 2A).

Plants contain three different isoforms of invertases, namely vacuolar (vac), cw-bound and neutral invertases, with different biochemical properties and sub-cellular localization [23]. Based on their pH optima, vac-Inv and cw-Inv are referred to as acidic soluble and insoluble invertases, respectively. We next investigated if the activity of vac-Inv was regulated by *Xcv* T3Es. As illustrated in Fig. 2B, there was approximately a two-fold increase in the activity of vac-Inv within 2 days after infection with either *Xcv* wild type or *Xcv* Δ*hrpB1* indicating that *Xcv* induces vac-Inv activity although to lower extent compared to that of cw-Inv. In contrast to cw-Inv, the activity of vac-Inv does not appear to be regulated by T3Es.

### Infection with *Xcv* Wild Type and *Xcv* ΔhrpB1 Causes Changes in the Amounts of Soluble Sugars and a Decreased Rate of Photosynthesis

**Figure 3:**
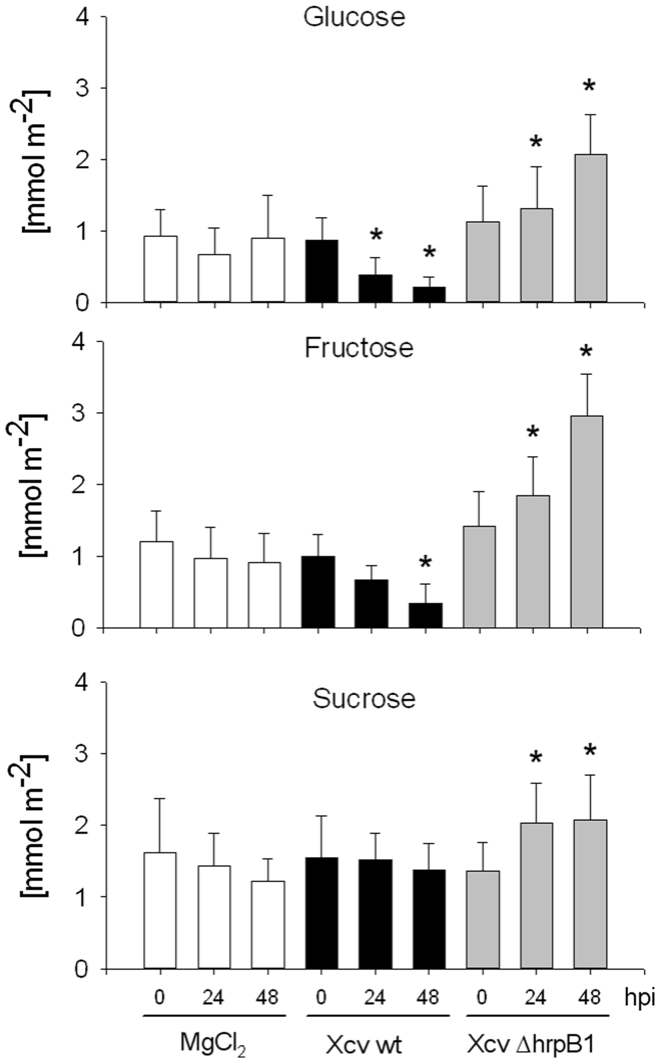
Content of soluble sugars in susceptible pepper leaves after infection with *Xcv* wild type or with the TTSS*-*deficient *Xcv* Δ*hrpB1* strain. Contents of glucose, fructose and sucrose were determined following inoculation of pepper leaves with *Xcv* wild type (wt) or the *Xcv* Δ*hrpB1* using a concentration of 5×10^8^ cfu ml^−1^ and compared to 10 mM MgCl_2_ infiltrated control leaves. Samples were taken before (0 h), 24 and 48 hours post infection (hpi). Each value represents the mean ± SE of four different experiments each with four to six individual samples. Statistically significant differences to Mock-inoculated control plants were determined using two-tailed t-test assuming normal distribution and are indicated by asterisks (*p<0.05).

Since an increase in cw-Inv activity upon infection with pathogens was found to be accompanied by an accumulation of soluble sugars [13], , we next determined the content of soluble sugars (*i.e.* glucose, fructose and sucrose) from total extracts of *Xcv* wild type- and *Xcv* Δ*hrpB1*-infected leaves. Sucrose content was not affected by *Xcv* wild type infection, but increased by 50% in response to infection with the T3SS-deficient *Xcv* Δ*hrpB1* strain (Fig. 3). The amount of glucose and fructose in pepper leaves decreased significantly following *Xcv* wild type infection by 48 h post-inoculation (Fig. 3). In contrast, there was an about 2-fold increase in the amount of hexoses after infection with *Xcv* Δ*hrpB1* (Fig. 3), which accompanied the strong increase in cw-Inv activity (Fig. 2A).

**Figure 4:**
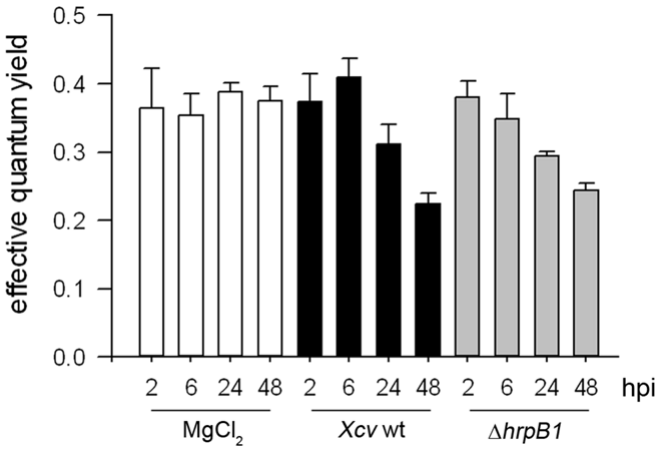
Changes in pepper leaf photosynthetic capacity after *Xcv* infection. Rate of photosynthesis, represented as effective quantum yield of photosystem II, was measured in susceptible pepper leaves 2, 6, 24 and 48 hours post infection (hpi) with the *Xcv* wild type strain (wt, black bars) and *Xcv* Δ*hrpB1* (grey bars*)* and compared to 10 mM MgCl_2_ (white bars) inoculated control leaves. Values represent the mean ± SE of four measurements performed with independent plants. The experiment was repeated twice with similar results.

Next we measured photosynthetic capacity in infected leaves because decreased amounts of *RbcS*-specific transcripts were detected after inoculation with *Xcv* wild type and *Xcv* Δ*hrpB1* (Fig. 1B). In accordance with the reduced expression of *RbcS*, the photosynthetic capacity (calculated as effective quantum yield) decreased after infection of pepper leaves with both *Xcv* wild type and *Xcv* Δ*hrpB1* reaching 60% and 64% of the initial values after 48 h (Fig. 4).

### Cw-Inv Induction is Mediated by *Xcv* PAMPs

Cw-Inv is known to be induced by PAMPs like chitosan and polygalacturonic acid or an elicitor preparation from the necrotrophic fungus *Fusarium oxysporus* [24], [25]. To investigate whether cw-Inv induction after *Xcv* infection is due to structural components that may act as PAMPs, cultures of *Xcv* wild type or *Xcv* Δ*hrpB1* were heat-inactivated and subsequently used for infiltration into pepper leaves.

For these and the following experiments, about 6-week-old susceptible pepper plants grown in the greenhouse were used for infections. Under these conditions, non-treated *Xcv* wild type induced clear disease symptoms after 3 days post infection (dpi). Samples were taken daily until 3 dpi. For control purposes, pepper leaves were infiltrated with 10 mM MgCl_2_.

**Figure 5:**
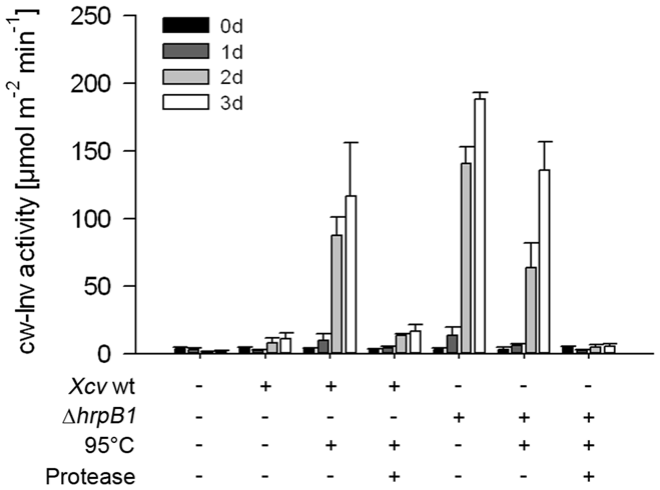
Cw-Inv activity of susceptible pepper leaves infected with heat-inactivated and protease-treated preparations of *Xcv* cells. *Xcv* wild type (wt) and *Xcv* Δ*hrpB1* were grown over night in NYG medium and prepared as described in material and methods. Subsequently, cells were heat-inactivated at 95°C for 20 min. An aliquot was of each cell culture was digested with proteases (proteinase K and trypsin) for 2 h at 60°C. After heat-inactivation for 10 min, cells were pelleted and re-suspended in 10 mM sterile MgCl_2_ and adjusted to OD_600_ = 1. Heat-inactivated *Xcv* cells with or without protease treatment were used for inoculation of pepper leaves. Samples were taken before (0 d), 1, 2 and 3 days post infections (dpi) and cw-Inv activity was measured. Values represent the mean of four independent samples ± SD. Similar results were obtained in an independent experiment.

Cw-Inv activity was strongly indΔuced in pepper leaves inoculated with heat-inactivated *Xcv* compared to pepper leaves inoculated with non-treated *Xcv* wild type (Fig. 5). The increase in cw-Inv activity brought about by inoculation with the heat-inactivated *Xcv* wild type strain was similar to those after infection with both alive and inactivated *Xcv* Δ*hrpB1* strains. These data confirm that cw-Inv activity is triggered by exposure to *Xcv* PAMPs. Moreover, they further suggest that *Xcv* T3E(s) suppress cw-Inv activity.

To test whether the cw-Inv elicitor activity is of proteinaceous nature, heat-inactivated *Xcv* preparations were treated with proteinase K and trypsin, which were applied simultaneously. After heat-inactivation of the proteases, *Xcv* cells were pelleted and re-suspended. Subsequently, the treated *Xcv* cells were infiltrated into pepper leaves and the effect on cw-Inv activity was determined (Fig. 5). Interestingly, the induction of cw-Inv activity due to infection with *Xcv* Δ*hrpB1* or the heat-inactivated *Xcv* wild type was abolished by protease treatment, indicating that the signal responsible for cw-Inv induction is likely a proteinaceous compound.

### Identification of Bacterial T3Es Involved in Suppression of cw-Inv Activity

The strong induction of cw-Inv mRNA abundance and enzyme activity after infection with the T3SS-deficient *Xcv* Δ*hrpB1* strain suggests that *Xcv* T3Es directly or indirectly regulate the function of cw-Inv in leaves. To begin to unravel the underlying mechanism, we aimed at identifying individual T3Es that significantly modulate cw-Inv enzymatic activity during *Xcv* infection. *Xcv* is predicted to utilize approximately 28 T3Es based on experimental data or bioinformatic analyses [5]–[7]. Eighteen different *Xcv* strains harbouring mutations in genes encoding T3Es were tested for their effect on cw-Inv activity upon infection and compared to the *Xcv* wild type response. Individual *Xcv* mutant strains were either generated within this study, by overlapping extension PCR and subsequent triparental mating, or have been generated and described in previous studies (Table 1). For the experiments, samples were taken before (0), 2 and 3 dpi, since clear differences in cw-Inv activity were detectable at these time points (Fig. 5). For control purposes, leaves were infiltrated with 10 mM MgCl_2_ and with *Xcv* Δ*hrpB1*.

**Table 1:** *Xanthomonas campestris* pv. *vesicatoria* strains used in this study.

Strain	Gene No.	Characteristics	Reference
*Xcv* 85-10, wild type		Pepper race 2, wild type strain 85-10, Rif^R^	[82]
*Xcv* Δ*hrpB1*	XCV0427	*hrpB1* deletion mutant of 85-10	[46]
*Xcv* Δ*hrpF*	XCV0411	*hrpF* deletion mutant of 85-10	[65]
*Xcv* Δ*avrBs2*	XCV0052	*avrBs2* deletion mutant of 85-10	This study
*Xcv* Δ*avrRxv*	XCV0471	*avrRxv* deletion and frameshift mutant of 85-10	This study
*Xcv* Δ*xopB*	XCV0581	*xopB* deletion mutant of 85-10	[70]
*Xcv* Δ*xopB* (Δ1257)	XCV0581	*xopB* deletion mutant of 85-10	This study
*Xcv* Δ*xopC1*	XCV2435	*xopC1* deletion mutant of 85-10	[34]
*Xcv* Δ*xopD*	XCV0437	*xopD* deletion mutant of 85-10	[83]
*Xcv* Δ*xopE1*	XCV0294	*xopE1* deletion mutant of 85-10	This study
*Xcv* Δ*xopE2*	XCV2280	*xopE2* deletion and frameshift mutant of 85-10	This study
*Xcv* Δ*xopF1*	XCV0414	*xopF1* deletion mutant of 85-10	[83]
*Xcv* Δ*xopF2*	XCV2942	*xopF2* deletion mutant of 85-10	This study
*Xcv* Δ*xopG*	XCV1298	*xopG* deletion mutant of 85-10	This study
*Xcv* Δ*xopJ*	XCV2156	*xopJ* deletion mutant of 85-10	[70]
*Xcv* Δ*xopN*	XCV2944	*xopN* deletion mutant of 85-10	[83]
*Xcv* Δ*xopO*	XCV1055	*xopO* deletion mutant of 85-10	[83]
*Xcv* Δ*xopP*	XCV1236	*xopP* deletion mutant of 85-10	[83]
*Xcv* Δ*xopQ*	XCV4438	*xopQ* deletion mutant of 85-10	[83]
*Xcv* Δ*xopX*	XCV0572	*xopX* deletion mutant of 85-10	This study
*Xcv* Δ*ecf*	XCV3785	*ecf* deletion mutant of 85-10	This study
*Xcv ΔxopAJ*	XCV4428	*xopAJ* deletion mutant of 85-10	This study
*Xcv* Δ*xopAK*	XCV3786	*xopAK* deletion mutant of 85-10	This study
*Xcv wt+pBBR1MCS5 EV)*		wild type strain 85-10 containing pBBR1MCS-5 plasmid, Rif^R^, Genta^R^	This study
*Xcv* Δ*xopB+pBBR1MCS5 EV)*		*xopB* deletion mutant of 85-10 containing pBBR1MCS-5 plasmid, Rif^R^,Genta^R^	This study
*Xcv* Δ*xopB+pBBR::xopB*(+/−)		*xopB* deletion mutant of 85-10 containing pBBR::xopB plasmid to restorewild type, Rif^R^, Genta^R^	This study
*Xcv* Δ*xopB* (Δ1257)*+pBBR1MCS5 EV)*		*xopB* deletion mutant of 85-10 containing pBBR1MCS-5 plasmid, Rif^R^,Genta^R^	This study
*Xcv* Δ*xopB* (Δ1257)*+pBBR::xopB*(−)		*xopB* deletion mutant of 85-10 containing pBBR::xopB plasmid to restorewild type, Rif^R^, Genta^R^	This study

**Figure 6:**
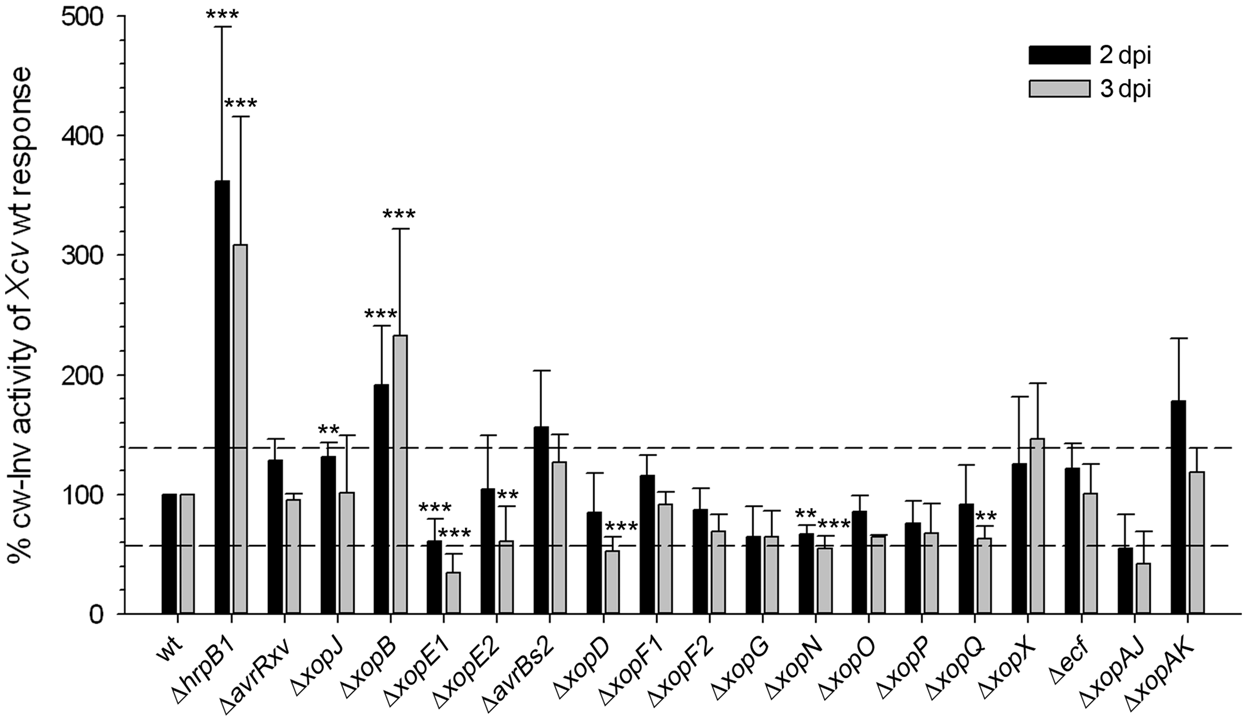
Screening for *Xcv* T3Es involved in regulation of cw-Inv activity. Leaves of pepper plants were infiltrated with wild type and mutant *Xcv* strains at 10^9^ cfu ml^−1^ and cw-Inv activity was measured 2 and 3 days post infection (dpi) in independent experiments. Graphs represent values calculated relative to the *Xcv* wild type (wt) response which was set to 100% for each individual experiment. Mean cw-Inv activities after infection with *Xcv* wild type were 20.96 µmol min^−1^ m^−2^±8.43 (100% ±40.2) and 57.57 µmol min^−1^ m^−2^±23.92 (100% ±41.6) at 2 and 3 dpi, respectively. The variance of *Xcv* wild type response (ca. 42%) is illustrated as a dashed line. Values are the mean response (as percentage to *Xcv* wild type) ± SD from three to nine different experiments. Statistically significant differences from *Xcv* wild type response were determined using two-tailed t-test assuming normal distribution and are indicated by asterisks (**p<0.01); (***p<0.001).

A typical set of data is shown in Fig. S1. As described before, cw-Inv activity was induced after infection with the *Xcv* wild type strain. However, the increase in cw-Inv activity was much stronger when T3SS-deficient strains (*Xcv* Δ*hrpB1* or *Xcv* Δ*hrpF*) were inoculated (Fig. S1). The different effector mutants were repeatedly tested in different sets of experiments. Due to the cultivation of plants in greenhouse there was a high variation (about 42%) in cw-Inv activity after *Xcv* wild type infection of pepper leaves over the different experiments. To enable comparison of individual data sets and to determine which T3E has a significant effect on cw-Inv activity, the cw-Inv activity was calculated upon infection with the individual *Xcv* mutant strains and the deviation to the cw-Inv activity caused by *Xcv* wild type was determined as percentage. The values obtained from up to nine different screenings were compiled and the mean (percentage) was calculated (Fig. 6). Only those T3E mutant strains that caused highly significant (p-value <0.01) changes compared to *Xcv* wild type were considered. Overall, seven *Xcv* T3E mutants showed a significantly different response 2 and/or 3 dpi compared to *Xcv* wild type infection: *Xcv* Δ*xopJ*, *Xcv* Δ*xopB*, *Xcv* Δ*xopE1*, *Xcv* Δ*xopE2*, *Xcv* Δ*xopD*, *Xcv* Δ*xopN*, *Xcv* Δ*xopQ* (Fig. 6). Five *Xcv* strains, namely those with mutations in *xopE1*, *xopE2*, *xopD*, *xopN* and *xopQ* caused a significant reduced activation of cw-Inv activity. Infection with *Xcv* Δ*xopE1* caused the most prominent effect leading to a 40% and 65% lower induction of cw-Inv activity compared to wild type after 2 and 3 days, respectively (Fig. 6). Infection with two *Xcv* T3E-deficient mutants led to an increased cw-Inv activity, namely *Xcv* Δ*xopJ* and *Xcv* Δ*xopB* (Fig. 6). Also inoculation with strains *Xcv* Δ*avrBs2*, *Xcv* Δ*xopX*, *Xcv* Δ*xopAK* tended to induce higher cw-Inv activity compared to *Xcv* wild type, but due to higher variation these changes are not statistically significant or are less significant (p-value <0.05) (Fig. 6, Fig. S1). Infection of pepper leaves with *Xcv* Δ*xopJ* only led to 30% higher cw-Inv activity compared to *Xcv* wild type 2 dpi, while *Xcv* Δ*xopB* infections elicited an increase in cw-Inv activity to 191% and 233% after 2 and 3 dpi, respectively. Overall, *Xcv* Δ*xopB* infections resulted in the strongest increase of cw-Inv activity compared to any other *Xcv* T3E mutant strain tested which led us to conclude that this effector plays a key role in suppression of cw-Inv activity. Therefore in further work, we focused on analysis of XopB.

### Effect of xopB Deletion on Gene Expression

**Figure 7:**
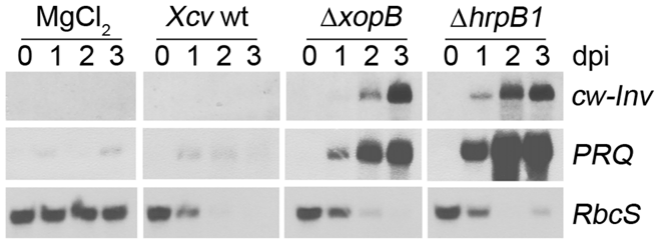
Expression of *cw-Inv*, *PRQ* and *RbcS* in susceptible pepper leaves in response to infection with *Xcv* Δ*xopB*. Leaves of pepper plants were infected with the *Xcv* wild type (wt), *Xcv* Δ*hrpB1*, *Xcv* Δ*xopB* using a concentration of 10^9^ cfu ml^−1^, and as control with 10 mM MgCl_2_.Total RNA was isolated from pepper leaves before (0), and 1, 2, 3 days post infection (dpi). Twenty five µg of total RNA was separated per each lane. Northern blots were hybridized with [^32^]P dCTP-labelled cDNA fragments of *cw-Inv*, *PRQ* and *RbcS.* A representative experiment is shown. Similar results were obtained in two other experiments.

To investigate whether XopB alters *cw-Inv* abundance at the transcriptional level, total RNA was isolated from pepper leaves before and 1–3 dpi with *Xcv* wild type, *Xcv* Δ*xopB*, *Xcv* Δ*hrpB1* or 10 mM MgCl_2_, and then transcript accumulation of *cw-Inv*, *PRQ*, and *RbcS* was analyzed by Northern blotting (Fig. 7). As expected, only a low level of *cw-Inv* and *PRQ* mRNA was detected in pepper leaves infected with *Xcv* wild type (Fig. 7 *).* In contrast, infection with either *Xcv* Δ*hrpB1* or *Xcv* Δ*xopB* resulted in high levels of *cw-Inv-* and *PRQ*- specific transcripts, which was more pronounced after *Xcv* Δ*hrpB1* treatment. *RbcS* mRNA abundance decreased after infection with all three *Xcv* strains and was hardly detectable at 2 and 3 dpi (Fig. 7).

To investigate whether XopB inhibits the expression of other defense-related genes, we analyzed the expression *Pti5* and *Acre31*. Both genes have been used as markers for PTI in tomato and/or *N. benthamina* and were shown to be significantly induced upon inoculation with T3SS-deficient strains [41], [42], [48]. To enable the analysis in pepper plants we used the published sequences of *SlActin* (AB199316.1), *SlPti5* (LEU89256) and *NtAcre31* (SGN-U434705) to search for orthologous sequences in pepper using the BLAST tool provided by the Solanaceae Genomic Network (http://solgenomics.net/). This led to the identification of the closely related sequences *CaActin* (SGN-U196461 *CaPti5* (SGN-U198861) and *CaAcre31* (SGN-U198671) which exhibit 89%, 86% and 83% identity, respectively to the query sequences.

**Figure 8:**
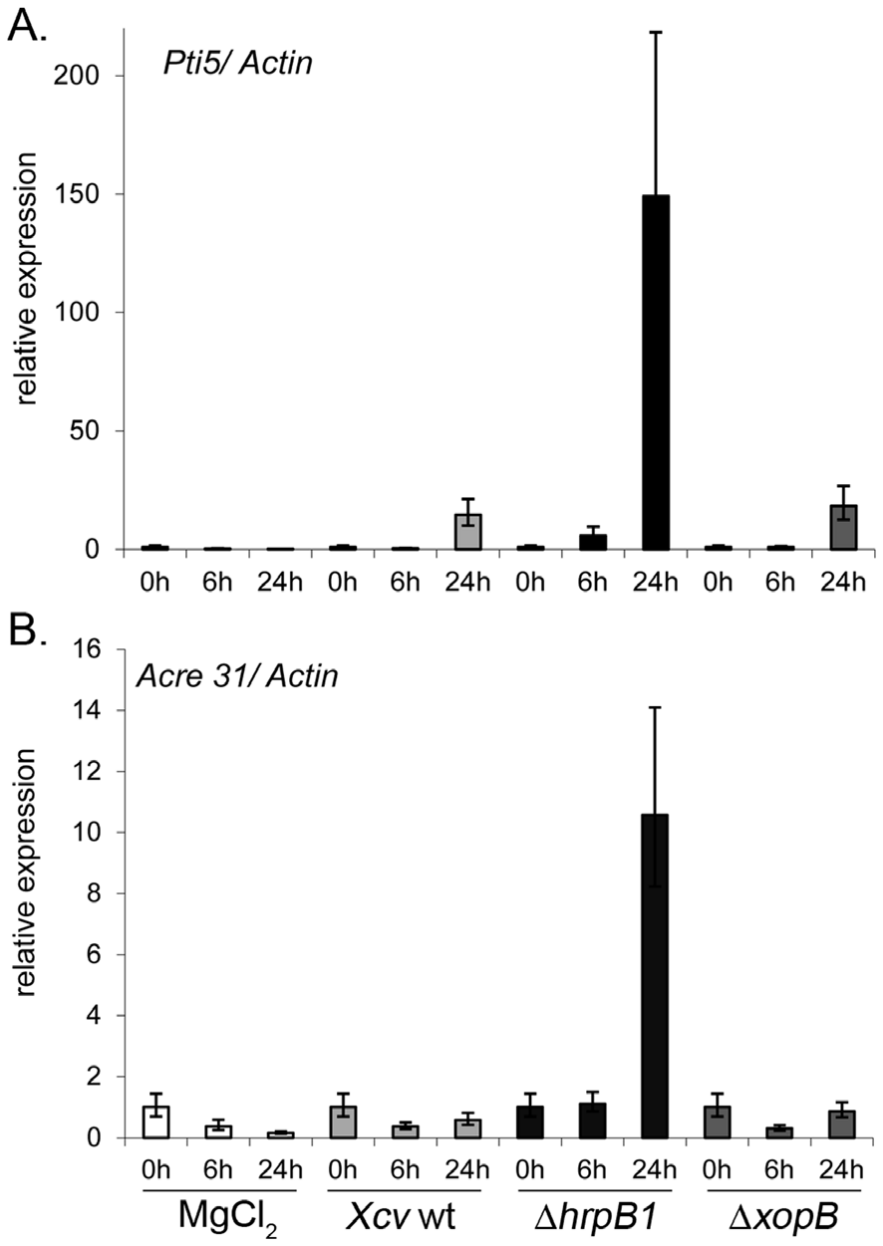
Expression of PTI marker genes *Pti5* and *Acre31* in susceptible pepper leaves after infection with different *Xcv* strains. Leaves of pepper plants were inoculated with *Xcv* wild type (wt), *Xcv* Δ*hrpB1*, *Xcv* Δ*xopB* using a concentration of 10^9^ cfu ml^−1^, and with 10 mM MgCl_2_. Total RNA was isolated from samples taken before (0 h), 6 h and 24 h after infiltration and reverse transcribed into cDNA. Abundance of *Pti5* (A.) and *Acre31* (B.) mRNA was detected by qPCR. Data were analysed using MxPro software v4.1. The expression levels of *Pti5* and *Acre31* were normalized with *Actin* and displayed relative to the expression level at time point 0 h which was set to a value of 1. The average ± SE of three replicates is shown. Similar results were obtained in an independent experiment. White bars, MgCl_2_; light grey, *Xcv* wild type; black, *Xcv* Δ*hrpB1*, dark grey, *Xcv* Δ*xopB*.

Total RNA was isolated from inoculated tissue at 6 h and 24 h after inoculation and relative abundance of specific mRNAs was ascertained by quantitative real-time PCR. Relative expression values for each treatment were compared to that obtained at time point “0 h” which was arbitrary set to 1 (Fig. 8). There was a large increase in *Pti5*- and *Acre31*- specific transcripts in pepper leaves 24 h after inoculation with *Xcv* Δ*hrpB1* compared to those infected with *Xcv* wild type (Fig. 8). This indicates that *Pti5* and *Acre31* mRNA abundance in pepper were induced in response to *Xcv* Δ*hrpB1* and that *Xcv* T3Es suppress their expression. Strikingly, infection with *Xcv* Δ*xopB* infection did not cause significant changes in *Pti5* or *Acre31* mRNA levels as compared to the response caused by *Xcv* Δ*hrpB1* infection (Fig. 8). This suggests that XopB does not affect the mRNA abundance of the two PTI-associated genes, but regulates the expression of *cw-Inv* and *PRQ* (Fig. 7).

### Complementation of the Effect of *Xcv* ΔxopB on cw-Inv Activity

**Figure 9:**
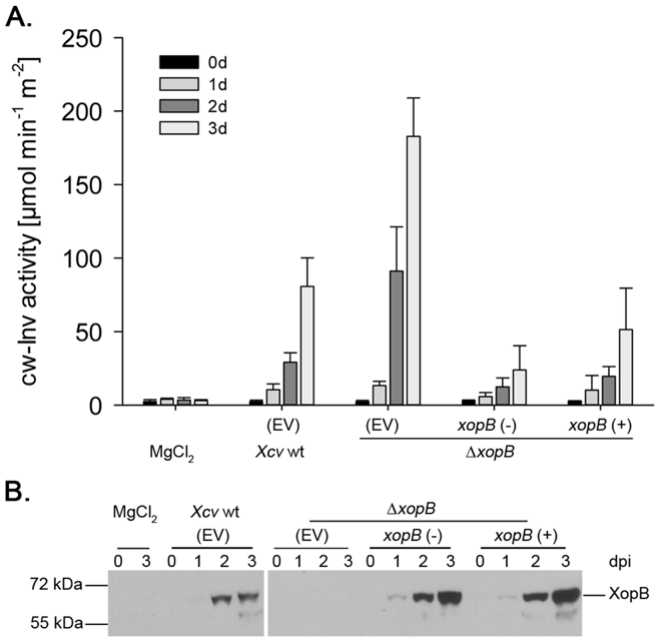
Expression of XopB in *Xcv* Δ*xopB* complements the effect on cw-Inv activity. Susceptible pepper leaves were inoculated with *Xcv* wild type (wt), *Xcv* Δ*xopB* containing both the pBBR1MCS5 vector (EV), or *Xcv* Δ*xopB* derivatives in which a genomic fragment was introduced containing the putative *xopB* promoter and open reading frame in sense (+) or antisense (−) orientation relative to the *lac* promoter. Samples were taken before (0), 1, 2 and 3 days post infection (dpi). A.) Cw-Inv activity was measured from the infected leaf tissue. Values represent the mean of four independent samples ± SD. B.) Expression of XopB was verified by Western blotting by probing with the anti-XopB antibody. XopB migrates at ∼70 kDa.

To confirm that suppression of cw-Inv is brought about by deletion of *xopB*, two complementation constructs were generated using the pBBR1MCS5 vector [49] that expresses the gene of interest under control of the *lac*Z promoter. To this end, a 2.6 kb genomic fragment encompassing the *xopB* region (−649 till 1949 bp) was amplified by PCR from the *Xcv* wild type strain (85-10). The fragment was cloned into the pBBR1MCS5 vector in both orientations referred to as pBBR::xopB(−) and pBBR::xopB(+). These constructs were conjugated into the *Xcv* Δ*xopB* mutant strain. The resulting *Xcv* strains were used for infection experiments and compared to the *Xcv* wild type and *Xcv* Δ*xopB* strains in which the empty vector was inserted. Activity of cw-Inv in pepper leaves was measured 1, 2, and 3 dpi. As expected, *Xcv* wild type infection resulted in the induction of cw-Inv activity (Fig. 9). However, the level of *Xcv-*induced cw-Inv activity was high compared to other experiments (Fig. 6, Fig. S1) reflecting the experimental variation detected under the conditions tested on different days. Nonetheless, the induction of cw-Inv activity in response to *Xcv* Δ*xopB* infection was higher than that triggered by *Xcv* wild type (Fig. 9A). Strikingly, the induction of cw-Inv activity by the *xopB* deletion strain could be overcome by introducing both plasmids. Thus, the cw-Inv activity after infection with *Xcv* Δ*xopB* containing the plasmid pBBR::xopB in either direction was comparable to that after *Xcv* wild type inoculation indicating that the effect of the *xopB* deletion could be complemented (Fig. 9A). The presence of XopB protein was confirmed by western blot analysis using an anti-XopB antibody that was generated against His-tagged XopB purified from *E. coli* (Fig. 9B). In the western blot, XopB protein expression could be detected upon infection with the *Xcv* Δ*xopB* strains containing either complementation construct. The presence of XopB could also be detected in pepper leaves after infection with the *Xcv* wild type strain, but not after inoculation of the *Xcv xopB* deletion strain (Fig. 9B).

To confirm these results, we generated an independent *xopB* deletion strain *Xcv* Δ*xopB* (Δ1257) (bp 259–1515) and complemented it with the pBBR::xopB(-) plasmid. Together with the *Xcv* wild type which harbours the empty vector, these strains were inoculated into pepper leaves and cw-Inv activity was measured (Fig. S2). Also this *xopB* deletion strain caused a strong increase in cw-Inv activity compared to *Xcv* wild type and this induction was abolished by introduction the complementation plasmid.

### Expression of xopB in Transgenic Tobacco Plants Suppresses Induction of cw-Inv Activity and Severely Impairs Plant Growth and Development

As an alternative approach to confirm that XopB suppresses cw-Inv activation, we aimed at expressing *xopB* in transgenic plants. To this end, the open reading frame coding for XopB was cloned and inserted between the CaMV 35S promoter and octopin synthase polyadenylation signal of the pBinAR vector [50] and the resulting plasmid was transformed into tobacco plants. Several transgenic lines could be regenerated which did not show obvious phenotypic changes in tissue culture. However after transfer into the greenhouse, the transgenic plants exhibited severe phenotypic alterations. Leaf development was significantly affected leading to malformed leaves and a dieback of the apical and lateral meristems (Fig. S3B). Expression of the *xopB* transgene was verified by Northern blot analysis of selected lines (Fig. S3A). Similar phenotypic changes were observed in transgenic tomato plants transformed with the same construct (Fig. S3C). These heavily impaired transgenic plants did not flower, were sterile and could consequently not be used for further experiments.

**Figure 10:**
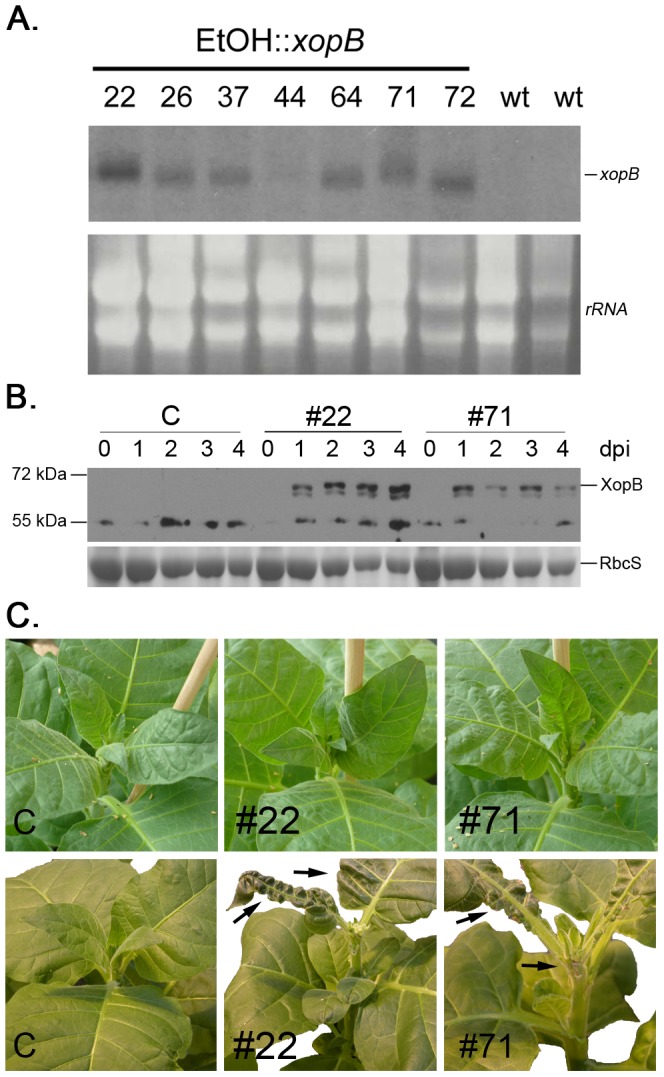
Inducible *xopB* expression in transgenic tobacco plants causes severe leaf abnormality. A.) Analysis of *xopB*-specific transcript accumulation in transgenic tobacco lines. Seven different lines (No. 22, 26, 37, 44, 64, 71, 72) and two control plants (wt) were analysed for *xopB* expression by Northern blotting. Total RNA was isolated 1 day after watering plants with 1% ethanol to induce *xopB* expression. Twenty µg of RNA were separated on a formaldehyde-containing agarose gel and analysed by hybridization with a *xopB*-specific radioactively labelled probe. Ethidium bromide stained rRNA is shown as loading control. B.) Analysis of XopB protein accumulation upon watering with 1% ethanol in selected transgenic lines (#22, #71). XopB migrates at ∼70 kDa, while in tobacco a cross-reactive band appeared at ∼55kDa. Expression of RubisCO as stained by Coomassie Blue is shown as control for protein loading. C.) Phenotypic changes in transgenic tobacco plants caused by *xopB* expression. Upper panel: symptoms 2 days after ethanol-treatment; lower panel: phenotypic alterations 10 days after induction. Arrows indicate morphological changes of the leaf lamina and cell death of meristematic tissue, respectively. From left to right: control line, lines #22 and #71.

To circumvent the problem caused by constitutive expression of *xopB in planta*, transgenic tobacco plants were generated that express *xopB* under the control of the ethanol-inducible promoter [51]. More than 70 transgenic plants were obtained which showed no morphological changes in a non-induced situation. To trigger expression of *xopB*, plants were watered with 1% (v/v) ethanol. Expression of the transgene was analyzed by Northern blotting of samples taken from young leaves 1 day after ethanol induction (Fig. 10A) and several positive lines were identified. Two transgenic lines (#22, #71) were selected for further analysis. Expression of the protein upon ethanol induction was verified in these lines by Western blotting using the XopB-specific antibody (Fig. 10B). The first phenotypic changes became visible in young, developing leaves 2 days after watering with ethanol (Fig. 10C). After 1 week, the morphological alterations of these leaves resembled those observed in transgenic plants with constitutive expression of *xopB*. However, normal new leaves could develop from unaffected lateral meristems and these plants produced normal flowers and seeds (Fig. 10C).

**Figure 11:**
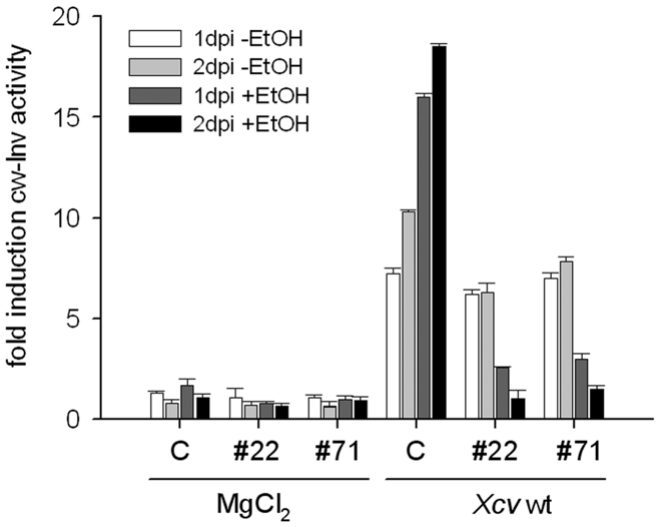
Inducible *xopB* expression in transgenic tobacco leaves suppresses cw-Inv activity during *Xcv* infection. Control plants and two selected transgenic tobacco lines with inducible *xopB* expression (#22, #71) were watered with 1% ethanol. After 24 h, plantlets were inoculated with a 10^9^ cfu ml^−1^ suspension of *Xcv* wild type. Samples were taken directly before inoculation and 1 and 2 days post inoculation (1dpi +EtOH; 2dpi *Xcv*+ EtOH). Non-ethanol watered plants were also inoculated with *Xcv* and samples were taken accordingly (1dpi -EtOH; 2dpi -EtOH). For control purposes ethanol-treated and non-treated plants were inoculated with 10 mM MgCl_2_. Cw-Inv activity was determined from four independent samples and fold changes ± SD were calculated for each sample relative to values obtained before *Xcv* inoculation. The experiment was repeated with similar results.

To study the impact of XopB on cw-Inv activity, the transgenic lines #22 and #71 were grown together with a transgenic control line which also harbors a kanamycin resistance gene [52]. One day after ethanol treatment, plants were infiltrated with *Xcv* wild type strain (OD_600_ = 1.0) or 10 mM MgCl_2_, and cw-Inv activity was measured 1 and 2 dpi. The induction was calculated for each treatment relative to the time point “0” (Fig. 11). Tobacco is a non-host plant for *Xcv* and a cell death response could be observed 3 to 4 dpi depending on greenhouse conditions (data not shown). In control plants, cw-Inv activity was strongly stimulated by *Xcv* infection at 1 and 2 dpi, but not by MgCl_2_ inoculation (Fig. 11). Interestingly, leaves expressing XopB only had a low level of cw-Inv activity during *Xcv* infection compared to the mock-inoculated transgenic plants. The cw-Inv activity only increased approximately 3-fold in both transgenic lines 1 day after *Xcv* infection, whereas in control plants the activity increased 16- to 18-fold. In contrast, cw-Inv activity was similar in *Xcv-*infected non-induced transgenic EtOH::xopB plants and control plants (Fig. 11). These data suggest that XopB suppresses the induction of host cw-Inv activity provoked by *Xcv* infection.

## Discussion

Until recently, little was known about the precise function of bacterial T3Es during plant pathogen interaction. The successful characterization of a number of effectors has provided the first insights into how they contribute to the pathogenic lifestyle of bacteria [8], [33], [36]. Almost two decades ago, it has been suggested that T3Es play an essential role in pathogenicity. In a very early study, Jakobek and colleagues showed that *Pseudomonas syringae* pv. *phaseolicola* has evolved strategies to suppress the expression of defense genes such as phenylalanine ammonia-lyase, chalcone synthase, chalcone isomerase and the production of phytoalexins in bean [53]. In addition, microscopic studies revealed that localized cell wall modification and associated papillae formation were caused by *Xcv hrp-*mutants but not by the wild type strain [54]. Using microarrays, Hauck et al. [55] showed that down-regulation of *Arabidopsis* genes involved in cell wall strengthening and plant defense by *P. syringae* DC3000 is caused by T3Es. More recent studies provided evidence that individual bacterial T3Es contribute to suppression of PTI and ETI responses, but only few are major virulence factors since deletion has only little effect on virulence (summarized in [36], [39]). Now the identification of their host targets and biochemical activities is the major issue to unravel the molecular basis of plant-bacteria interaction.

Sugars were suggested to contribute to immune responses against pathogens [20], [27], [47], [56]. It is now clear that the sucrose-to-hexose ratio in the apoplastic space has emerged as an important parameter linked to plant immunity which is largely controlled by cw-Inv. In most cases, cw-Inv mRNA expression and enzyme activity are induced at the infection site by diverse pathogens including fungi, oomycetes, viruses and bacteria [18]–[22]. We show that cw-Inv activity is increased in susceptible pepper leaves after infection with *Xcv* wild type but this was not associated with increased cw-Inv transcript abundance. The obvious discrepancy between increased cw-Inv enzyme activity and mRNA accumulation may be explained by at least two mechanisms. Firstly, protein turnover may be decreased leading to an increase in the cw-Inv protein amount relative to the mRNA level. Secondly, proteinaceous cw-Inv inhibitor proteins have been discussed in numerous plants species, although their molecular mode of action and their regulation are still under debate [57], [58]. Nevertheless, they may be responsible for the *in planta* modulation of cw-Inv activity. The second hypothesis is in accordance with a report from Bonfig et al. [59].

In a first work, Bonfig et al. reported an accumulation of *cw-Inv1* transcript after infection of *A. thaliana* with *P. syringae* DC3000 but this was not paralleled by an increased enzyme activity [60]. However, an elevated cw-Inv activity was demonstrated using histochemical activity stain in a subsequent study [59]. The difference in the measurable cw-Inv activity was explained by the release of cw-Inv enzyme from repression by the cw-Inv invertase inhibitor. Accordingly, the expression of the invertase inhibitor At *C/VIF2* was shown to be down-regulated after *P. syringae* DC3000 infection of *A. thaliana* plants [59].

Cw-Inv activity is induced by several stimuli including sugars and PAMPs (*e.g.* chitosan and a crude elicitor preparation of the fungus *Fusarium oxysporum lycopersici*) [24]. In our study, heat-inactivated *Xcv* cells triggered a strong induction of cw-Inv activity and this induction was abolished by protease-treatment suggesting that proteinaceous compounds or structures from *Xcv* are most likely the stimulus. Both lipopolysaccharides and the peptide-bearing peptidoglucans from *Xanthomonas* ssp. have been shown to act as PAMPs and elicit basal defense responses [61], [62]. The most extensively studied bacterial PAMPs are the small peptides flg22 and elf18 derived from flagellin and the translation elongation factor EF-Tu, respectively. However to date, the respective PAMPs from *Xcv* have not been associated with a stimulation of PTI in solanaceous plants. While flagellin from some *X. campestris pv. campestris* strains were shown to elicit FLS2-dependent defense responses in *A. thaliana* [63], a crude flagellin preparation from *Xcv* did not stimulate a defense response in tomato cells [64]. Recent publications suggest that conserved components of the T3SS machinery itself may serve as PAMPs that are specifically recognized by receptors to trigger plant immune responses ([39] and references therein). Thus, further experiments are necessary to elucidate the identity of the cw-Inv inducer from *Xcv*.

Infection of pepper plants with the T3SS-deficient strain *Xcv* Δ*hrpB1* resulted in a progressive increase in cw-Inv mRNA abundance, which was accompanied by a strong increase in cw-Inv enzyme activity compared plants infected with *Xcv* wild type. This result led us to conclude that *Xcv* T3Es are likely involved in the suppression of cw-Inv transcription and enzyme activity. Using the *Xcv* Δ*hrpF* mutant lacking the translocation apparatus [65], we could show that delivery of T3Es into plant cells is required to achieve suppression of cw-Inv. Moreover, the elevated cw-Inv activity upon inoculation with the T3SS-deficient strain was paralleled by an accumulation of soluble sugars, while infection with *Xcv* wild type led to reduced amounts of glucose and fructose. In the latter case, the reduced level of hexoses may be due to bacterial uptake and consumption and/or to a lower accumulation of sugars due to metabolic disturbances. A decrease in the rate of photosynthesis and a reduction in the photosynthesis-related *RbcS* transcript were observed in the *Xcv-*infected pepper leaves. *Xcv* wild type infection causes macroscopically visible disease symptoms (*i.e.* chlorosis and necrosis) in pepper leaves. As the pathogen multiplies and colonizes its host tissue, it is hypothesized that the pathogen re-directs plant metabolism to allocate nutrients for its own benefit. The outcome of this interaction results in metabolic perturbance in the plant cell as well as host cell leakage which are most likely responsible for a rapid decline in photosynthesis. This is in accordance with the decline in photosynthesis observed after infection with several pathogens (summarized in [30]).

Notably, a decreased rate of photosynthesis and *RbcS* mRNA expression was also observed after infection with the *Xcv* Δ*hrpB1* strain. In this case, bacterial propagation was restricted due to PTI and no disease symptoms were apparent at 2 dpi. The decrease in photosynthetic activity cannot be explained by cell damage caused by bacteria. It could however be due to the cw-Inv dependent generation of sugar signals in the apoplast. In addition to their classical role as a source of carbon and energy, soluble sugars act as signaling molecules and can regulate gene expression [29], [66]. An accumulation of soluble sugars, in particular hexoses, is known to down-regulate photosynthetic gene expression [29], [30], [66] and to increase expression of defense-related genes, such as *PRQ* [47], [66]. Thus, hexoses released by cw-Inv activated during *Xcv* Δ*hrpB1* infection of pepper leaves may act as signalling molecules to repress photosynthetic gene expression and to increase *PRQ-*specific transcript abundance. This assumption is supported by an earlier study in which cw-Inv activity was required to induce senescence-associated and *PR-* genes (including *PRQ*) after *Xcv* infection and was linked to a down-regulation of photosynthetic activity and *RbcS* mRNA abundance [21]. The results indicate that suppression of cw-Inv may contribute to a longer maintenance of photosynthesis activity and dampening of basal defense. Together, our results suggest that T3Es suppress the induction of cw-Inv activity to minimize the generation of sugar signals in infected plant leaves.

In order to identify *Xcv* T3E(s) involved in regulation of cw-Inv activity, we performed a screen with eighteen *Xcv* mutants deficient in individual effectors. Among them, several *Xcv* deletion strains (*e.g. Xcv* Δ*xopE1*, *Xcv* Δ*xopE2*, *Xcv* Δ*xopD*, *Xcv* Δ*xopN*) suppressed cw-Inv activity in peppers leaves relative to that detected in *Xcv* wild type-infected leaves. This was unexpected from the strong induction caused by T3SS-deficient strains, and may argue for tight regulation of cw-Inv with T3Es acting as activators and suppressors. Hence, they might contribute to activation of cw-Inv activity during later stages of infection.

T3Es acting at the plasma membrane could potentially influence secretion or activity of cw-Inv. XopE1 and XopE2 were shown to harbor a conserved N-myristoylation motif that is most likely responsible for targeting the proteins to the plasma membrane [67]. Using *xopE1* and *xopE2* deletion strains, no differences in disease and HR development could be detected when compared to *Xcv* wild type [67]. They belong to the HopX effector family, which is part of the transglutaminase superfamily encompassing proteins with different enzymatic activities (e.g. proteases; see [67]), but the enzymatic activity of XopE1 and XopE2 is unclear. In our screen, they appeared to promote cw-Inv activity, which needs to be secreted into the extracellular milieu. XopN is involved associated with the host plasma membrane and involved in the suppression of PTI. The *Xcv* Δ*xopN* mutant was shown to be impaired in bacterial growth and development of disease symptoms in tomato leaves [41]. This delay may also contribute to a weaker induction of cw-Inv activity. In contrast, tomato leaves infected with *Xcv* Δ*xopD* developed disease symptoms like chlorosis and necrosis faster than *Xcv* wild type infected plants [68]. The authors speculated that XopD may contribute to multiplication of *Xcv* during later stages of infection by suppressing leaf senescence and symptom development. It was shown that XopD localizes to subnuclear foci and exhibits a DNA-binding domain that may regulate transcription of target genes [68], [69].

In contrast, infection with *Xcv* Δ*xopJ* or *Xcv* Δ*xopB* resulted in increased cw-Inv activity at 2 and/or 3 dpi. XopJ is a member of YopJ/AvrRxv family of T3Es with SUMO peptidase and acetyltransferase activity. Like XopE1 and XopE2, it localizes to the plasma membrane which is most likely mediated by an N-terminal N-myristoylation motif [43], [67]. Furthermore, it could be demonstrated that expression of XopJ in *N. bethamiana* inhibits secretion of a secGFP marker and that transgenic *A. thaliana* plants with inducible expression of XopJ are compromised in their ability to deposit callose. These data led to the suggestion that XopJ interferes with vesicle transport and thereby with cell wall-based defense [43]. Since cw-Inv is also a protein that needs to be secreted, the higher cw-Inv activity measured 2 days after infection with the *Xcv* Δ*xopJ* strain may be explained by the negative influence of XopJ on protein secretion. The impact on cw-Inv may be overcome by other effectors which may have a redundant function during later stages of infection.

The most prominent effect on cw-Inv was seen after infection with *Xcv* Δ*xopB*. XopB was identified as a HrpG controlled gene via a cDNA-AFLP screen and was shown to be secreted by the T3SS [70], [71]. The XopB protein consists of 613 amino acids and sequence comparisons revealed high sequence similarity only to proteins of phytopathogenic bacteria such as to HopD1 from *Pseudomonas syringae* pv. *tomato* and AvrPphD of *P. syringae* pv. *phaseolicola*, but nothing is known about its biological activity. As observed earlier by Noel et al. [70], inoculation of *Xcv* Δ*xopB* in susceptible pepper plants revealed no significant differences in timing of symptom development and bacterial growth compared to *Xcv* wild type, while in a very recent study, Schulze et al. [71] found that deletion of *xopB* led to reduced disease symptoms, although multiplication was not impaired compared to *Xcv* wild type. The different phenotypes in the mentioned studies were attributed to different environmental conditions. Deletion of *xopB* did not however influence the ability to elicit an HR in resistant pepper plants [71].

Using a protoplast assay, Schulze et al. [71] showed recently that XopB suppresses flg22-mediated induction of the *NHL10* (NDR1/Hin1-like10) promoter fused to luciferase which served as a reporter for basal defense. However, XopB did not interfere with flg22-triggered activation of MAPKs [71]. Here, we showed that XopB is a key regulator for the induction of cw-Inv and affects both its transcript abundance and its activity. Furthermore, XopB is involved in the suppression of *PRQ* gene expression. *PRQ* expression was found to be induced by sugars and salicylic acid, but sugar-mediated regulation was independent of salicylic acid [20], [47]. In contrast, XopB activity did not influence the mRNA abundance of *Pti5* and *Acre31*, two genes associated with PTI in tomato [41], [42], [48]. This indicates that XopB inhibits a specific set of defense genes including sugar responsive ones.

Schulze et al. [71] concluded from localization studies that XopB is confined to vesicle-like structures in the cytoplasm of the host cell and may inhibit intracellular vesicle trafficking. Vesicle trafficking which is important for correct localisation of PAMP receptors as well as for the export of PR proteins and other extracellular enzymes (like cw-Inv), and antimicrobial peptides appears to be an important part of plant immunity and some T3Es (*e.g.* XopJ and HopM1) were shown to target the secretion pathway [43], [72]. However compared to *Xcv* Δ*xopB*, deletion of *xopJ* from the *Xcv* genome had only a weak effect on cw-Inv in our assays. Whether XopB regulates directly or indirectly cw-Inv by inhibition of protein secretion or by a different mechanism remains to be elucidated.

Ectopic expression of XopB in transgenic tomato and tobacco plants caused severe phenotypic alterations including the appearance of cell death, in particular in young, growing tissue, which finally led to a dieback of meristems. Expression of XopB in yeast cells also resulted in growth inhibition [45] and it caused cell death when transiently expressed in *N. benthamiana*, but not in *N. tabacum* or tomato [45], [71]. Thus, XopB may interfere with processes that are important for plant development and plant immunity.

In summary, we could show that induction of cw-Inv in pepper plants is suppressed by *Xcv* T3Es with XopB as the key regulator. The suppression of cw-Inv most likely prevents generation of hexoses and thereby the down-regulation of photosynthesis and sugar-enhanced defense. However, the interrelation between XopB, cw-Inv and sugar-mediated defense response needs to be unravelled by further studies. Towards this aim, the identification of host target proteins will be an important step.

## Materials and Methods

### Bacterial Strains and Growth

Bacterial strains used in this study were as follows: *Escherichia coli* DH5 Δpir, XL1 Blue and M15, *Agrobacterium tumefaciens* C58C1 carrying the pGV2260 virulence plasmid [73] and *Xanthomonas campestris* pv. *vesicatoria* (*Xcv*) strain 85-10. All modified *Xcv* strains used in this study are described in Table 1. *E. coli* were grown on Luria Broth (LB) medium at 37°C, *A. tumefaciens* on yeast-beef extract-peptone (YEB) medium (supplemented with 0.5 g l^−1^ sucrose and 2 mM MgSO_4_) at 28°C containing appropriate antibiotics. *Xcv* strains were cultivated at 30°C on nutrient-yeast-glycerol (NYG) medium supplemented with antibiotics. Antibiotics were added to the media at following final concentrations: ampicillin, 166 µg ml^−1^; kanamycin, 50 µg ml^−1^; tetracyclin, 10 µg ml^−1^; rifampicin, 100 µg ml^−1^ (NYG), 50 µg ml^−1^ (LB and YEB); gentamycin 15 µg ml^−1^.

### Plant Material, Growth Conditions and Inoculation Experiments

For initial experiments susceptible pepper plants (*Capsicum annuum* cv. Early Cal Wonder (ECW)) were cultivated in a growth cabinet with 16 h light (250 µmol quanta m^−2^ sec ^–1^) and 8 h darkness. The temperature regime followed the day/night cycle with 28°C and 22°C, respectively, the relative humidity was between 70% and 80%.

For the effector screening and experiments to characterize *Xcv* Δ*xopB*, pepper plants were grown in greenhouse at 26°C with 16 h supplemental light (150–200 µmol quanta m^−2^ sec ^–1^) and 50–60% relative humidity. Tobacco plants (*Nicotiana tabacum* cv. Samsun NN) were grown in tissue culture under a 16 h light/8 h dark regime at 50% relative humidity on Murashige Skoog medium containing 2% (w/v) sucrose. Transgenic plants were selected on kanamycin in tissue culture and further cultivated in the greenhouse with 16 h supplemental light (150–200 µmol quanta m^−2^ sec ^–1^) at 25°C and at 22°C during 8 h of darkness. Expression of *xopB* was induced by watering individual pots with 50 ml 1% (v/v) ethanol.

*Xcv* strains were infiltrated at the abaxial side of fully expanded leaves of 5–6 week-old plants using a needless syringe. *Xcv* strains were grown over night in NYG medium. Bacteria were harvested by centrifugation at 5000 *g* for 20 min at 4°C, washed with 10 mM sterile MgCl_2_, followed by a second centrifugation step. The bacterial cells were re-suspended in 10 mM MgCl_2_ and adjusted to a final concentration of 5×10^8^ cfu ml^−1^ or 10^9^ cfu ml^−1^ (OD_600_ = 1).

### Bacterial Elicitor Preparation

*Xcv* stains grown over night in NYG medium were prepared as described above and adjusted to OD_600_ = 1. Cells were heat-inactivated by incubation at 95°C for 20 min in a water bath. An aliquot was subjected to treatment with proteinase K (Sigma-Aldrich) and trypsin (Sigma-Aldrich) at 0.2 mg ml^−1^ for 2 h at 60°C. After heat-inactivation for 10 min, cells were pelleted and re-suspended in 10 mM sterile MgCl_2_ to give an OD_600_ = 1. Heat-inactivated *Xcv* cells with or without additional proteinase K and trypsin treatment were used for infection experiments.

### Construction of *Xcv* Deletion Mutants

To generate *Xcv* deletion mutants primers listed in Table S1 were used. Gene deletions were introduced by overlap extension PCR using genomic DNA from *Xcv* wild type strain 85-10 as template. DNA fragments were cloned into pGEM-T easy (Promega, Madison, USA) and sequenced. Fragments were excised using added restriction sites (see Table S1) and cloned into the pOK vector. Plasmids were introduced into *Xcv* by conjugation using pRK2013 as helper plasmid in triparental matings as described by Huguet et al. [74].

### Construction of *Xcv* Δ*xopB* Complementation Plasmids

To complement *Xcv* Δ*xopB* [70] and *Xcv* Δ*xopB* (Δ1257 a genomic fragment was amplified by PCR from DNA of *Xcv* (85-10) using the primers given in Table S1. The fragment (−649 to +1949) was cloned into pCR blunt vector (Invitrogen, Karlsruhe, Germany) and sequenced. Subsequently, a 2.6 kB fragment was excised using *Bam*HI restriction sites and inserted into the broad host range vector pBBR1MCS5 [49] creating pBBR::xopB(+) and pBBR::xopB(−) which harbor the fragment in sense (5′ - 3′) or antisense orientation relative to the *lacZ* promoter. The plasmids were moved into *Xcv* Δ*xopB* strains by triparental mating.

### Generation of Transgenic Tobacco Plants

The open reading frame of *xopB* (accession no. AY036109) was amplified from genomic DNA of *Xcv* strain 85-10 using the gene-specific primers xopB_5′ GTC GAC AAC AAT GAA GGC AGA GCT CAC ACG ATC C and xopB_3′ GGA TCC TTA CGG CTC AGG CGC GGG TTG GTG. The resulting PCR fragment was sub-cloned into the pCR blunt vector (Invitrogen, Karlsruhe, Germany). The *xopB* fragment was excised using the *Sal*I/*Bam*HI restriction sites and inserted into a pUC-based plasmid between a chimeric *alcA* promoter and a *35S* terminator [51], [75]. The resulting *alcA* expression cassette was excised and inserted into pBin19-derived vector p35S::alcR using the *Asc*I restriction sites yielding the construct EtOH::*xopB*. Stable transformation of tobacco plants was performed by *Agrobacterium-*mediated gene transfer as described previously [76].

### RNA Isolation and Northern Blot Analysis

Isolation of total RNA was performed as described in Logemann et al. [77]. For Northern blot analysis 20–30 µg of total RNA were separated on 1.5% formaldehyde containing agarose gels and blotted onto nylon membrane (GeneScreen, NEN, Boston, USA) by capillary blotting overnight. The membranes were pre-hybridized and hybridized at 65°C. cDNA fragments of pathogenesis-related protein Q (PRQ) (Acc. No. X54456), cell wall invertase (cw-Inv) (Acc. No. X81834), cytosolic glyceraldehyde 3-phosphate dehydrogenase (GAPDH) (Acc. No. AF527779) and ribulose-1,5-bisphosphate carboxylase small subunit (RbcS) (Acc. No. X02353) were used as probes and radioactively labeled with [^32^P] dCTP by means of the High Prime Kit (Roche, Basel, Switzerland). After stringent washing, radioactive membranes were exposed to X-Ray films (Kodak) over night at −70°C.

### Enzyme Activity Assays

Leaf discs (0.5 cm^2^) were homogenised with 50 mM Tris buffer, pH 6.8, containing 5 mM MgCl_2_, 5 mM *β-*mercaptoethanol, 1 mM EDTA, 1 mM EGTA, 15% (v/v) glycerol and 0.1 mM Pefabloc proteinase inhibitor. The extracts were centrifuged for 10 min at 13,000 rpm at 4°C. An aliquot of the supernatant was desalted by centrifugation through Sephadex G-25 medium equilibrated in extraction buffer. The desalted extract was used for measurement of vacuolar (vac) invertase activity as described in [21]. Protein concentrations were determined using the Bio-Rad protein assay. The pellet was washed twice with 5 mM Tris buffer, pH 7.0, and centrifuged for 10 min at 13,000 rpm and 4°C. The activity of cell wall invertase (cw-Inv) was determined by resolving the pellet in 10 mM sodium acetate buffer, pH 5.0, containing 0.1 M sucrose and incubating the mixture at 37°C for 90 min. The mixture was neutralized by adding an aliquot of 1 M Tris-HCl, pH 8.0, and subsequently heat-inactivated at 95°C for 5 min. The amount of glucose formed was measured as described in [78].

### Determination of Soluble Sugars

Leaf discs (0.5 cm^2^) were extracted with 0.5 ml 80% (v/v) ethanol and incubated at 80°C for 90 min. After centrifugation at 4°C for 5 min at 13,000 rpm, cleared supernatants were transferred into new tubes and evaporated to dryness at 40°C. The residue was resolved in 250 µl water. An aliquot was used for determination of soluble sugars using an enzyme-coupled assay as described in [78].

### Chlorophyll Fluorescence

Chlorophyll fluorescence was measured using a PAM-2000 portable fluorometer (Walz, Effeltrich, Germany). After removing plants from the growth chamber leaves were quickly exposed to 750 µmol quanta m^−2^s^−1^ white light for 10 min before the maximum fluorescence yield (Fm′) was measured during a 0.8 sec pulse of white light with an intensity of 3,500 µmol quanta m^−2^s^−1^. The used light intensity of 750 µmol quanta m^−2^s^−1^ was close to light saturation of photosynthesis. Effective quantum yield of PSII in the light (Ψ_PSII_) was calculated according to the equation: ψ_PSII_ = (F_m_′–F_s_) : F_m_′ where F_s_ is the steady state fluorescence in the light adapted state.

### Generation of XopB Antibody and Immunoblotting

To obtain recombinant His_6_-tagged XopB, the coding region of *xopB* was amplified by PCR using the primers FP_xopB 5′-GGATCCAAGGCAGAGCTCACACGAT-3′ and RP_xopB 5′-GTCGACTTACGGCTCAGGCGCGG-3′ and cloned into pQE-9 (Qiagen, Hilden, Germany) using *Bam*HI and *Sal*I restriction sites. The construct was transformed into *E. coli* M15 (pRep4) cells. His_6_:XopB protein expression was induced at OD_600_ = 0.7 with 1 mM IPTG at 37°C and 200 rpm. Four hours after induction 1.2 g cells were harvested and lysed under denaturating conditions with 8 M urea, 100 mM NaH_2_PO_4_ and 10 mM Tris-HCl at pH 8.0. His_6_:XopB protein was purified from soluble cell extract with Ni-NTA agarose according to manufacturer’s instructions (Qiagen). For further purification, His_6_:XopB was subjected to preparative polyacrylamide gel electrophoresis, and the corresponding protein band of His_6_:XopB was eluted with an electro eluter (BioRad). For antibody production, 1.43 mg of pure His_6_:XopB solved in 25 mM Tris-HCl, 192 mM glycine and 0.1% SDS, pH 8.2 were used for immunization of rabbits (Biogenes, Berlin, Germany). The serum was further purified using affinity chromatography and used as primary antibody (dilution 1∶1000) in further analyses.

For Western blot analyses, leaf discs (0.5 cm^2^) were homogenised in 2× Laemmli buffer containing 126 mM Tris-HCl, pH 6.8, 20% glycerol, 4% SDS and 0.02% bromophenol blue. Extracts were incubated at 95°C for 10 min and centrifuged for 1 min at RT. 10–30 µg protein were separated on 12.5% (v/v) SDS-containing polyacrylamide gels. Proteins were transferred onto nitrocellulose membranes (Porablot NCL, Macherey-Nagel, Düren, Germany), blocked for at least 1 h in 5% skim milk/TBST (20 mM Tris, 500 mM NaCl, 0.1% (v/v) Tween 20) and incubated with anti-XopB antibody for 1 h at RT. The signal was detected following incubation with secondary peroxidase-conjugated antibody using the ECL-system (Thermo Scientific) according to manufacturer’s instruction.

### Quantitative RT-PCR

Quantitative real-time PCR (qPCR) was performed with cDNA and gene-specific primers. cDNA synthesis and qPCR were essentially performed as described by [79]. One µl of 1∶5 or 1∶10 diluted cDNA of each sample were amplified in three technical replicates on a Mx3000PQ-PCR system (Agilent Technolgies) in combination with the Brilliant II SYBR Green Q-PCR Master Mix Kit (Agilent technologies). Primers for the amplification of targets were taken from Kim et al. [41] for *Pti5,* Mason et al. [80] for *Actin* or designed using Primer3plus software [81] and were as follows: *Pti5* (LEU89256) forward primer 5′-ATTCGCGATTCGGCTAGACATGGT-3′, reverse primer 5′-AGTAGTGCCTTAGCACCTCGCATT-3′; *Acre31* (SGN-U198671) forward primer 5′-AGAGCCTCGAAATCGTCAAA-3′, reverse primer 5′- TGATGAACTCAGCCAAGCAC-3′ and *Actin* (AB199316) forward primer 5′- TAATCCCAAGGCCAACAGAG -3′ and reverse primer 5′- GAAAGCACAGCCTGGATAGC -3′. For each primer pair the efficiency was determined which were 150% (r^2^ = 0.970) for *CaActin*, 110% (r^2^ = 0.971) for *CaPti5* and 99.2% (r^2^ = 0.999) for *CaAcre31.* A melting analysis was performed at the end of each run to ensure that unique products were formed.

## Supporting Information

Figure S1 **Cw-Inv activity in pepper leaves after infiltration with** ***Xcv*** **wild type and** ***Xcv*** **mutant strains at a concentration of 10^9^ cfu ml^−1^.** Values represent the mean ± SD from two different experiments each with four independent samples. Black bars: before infection. Light grey: 2 days post infection (dpi). Dark grey: 3 dpi.
(TIF)

Figure S2 ***Xcv*** **Δ** ***xopB*** **(Δ1257) induces a strong induction of cw-Inv activity which is complemented by plasmid-borne expression of XopB.** Susceptible pepper leaves were inoculated with *Xcv* wild type (wt), *Xcv* Δ*xopB* (Δ1257) containing both the pBBR1MCS5 vector (EV), or a *Xcv* Δ*xopB* (Δ1257) derivative in which a genomic fragment was introduced containing the putative *xopB* promoter and open reading frame in antisense (−) orientation relative to the *lac* promoter. Samples were taken before (0) and 1, 2, 3 days post infection (dpi). A.) Cw-Inv activity was measured from the infected leaf tissue. Values represent the mean of four independent samples ± SD. B.) Expression of XopB was verified by Western blotting by probing with the anti-XopB antibody. XopB migrates at ∼70 kDa.
(TIF)

Figure S3 **Constitutive expression of** ***xopB*** **in transgenic tobacco and tomato plants.** The *xopB* open reading frame was inserted into the binary vector pBinAR between the CaMV 35S promoter and the polyadenylation site of the octopin synthase from *Agrobacterium tumefaciens* and transformed into tobacco and tomato plants. A.) Analysis of *xopB*-specific mRNA accumulation in transgenic tobacco lines. Total RNA was isolated from six different transgenic lines and from two wild type plants (wt). Expression of *xopB* using a gene-specific probe was verified by Northern blotting. B.) Phenotypic changes in transgenic tobacco plants caused by constitutive *xopB* mRNA expression. Shown are three independent lines compared to wild type (upper left panel). C.) Phenotypic changes in transgenic tomato plants caused by constitutive *xopB* expression. Shown are wild type (upper left panel) and several transgenic lines with different level of *xopB* expression.
(TIF)

Table S1 **Primers used for generation of different** ***Xcv*** **strains. Added restriction sites were marked by bold letters.**
(DOCX)

## References

[1] Stall RE (1995) *Xanthomonas campestris* pv. *vesicatoria*. In: Singh RP, Singh US, Kohmoto K, editors. Pathogenesis and host-parasite specificity in plant diseases. Tarrytown, New York: Pergamon, Elsevier Science Inc. 167.

[2] Büttner D, Bonas U (2010) Regulation and secretion of *Xanthomonas* virulence factors. FEMS Microbiol Rev 34: 107–133. 19925633 10.1111/j.1574-6976.2009.00192.x

[3] Büttner D, Bonas U (2002) Port of entry–the type III secretion translocon. Trends Microbiol 10: 186–192. 11912026 10.1016/s0966-842x(02)02331-4

[4] Alfano JR, Collmer A (2004) Type III secretion system effector proteins: double agents in bacterial disease and plant defense. Annu Rev Phytopathol 42: 385–414. 15283671 10.1146/annurev.phyto.42.040103.110731

[5] Thieme F, Koebnik R, Bekel T, Berger C, Boch J, et al (2005) Insights into genome plasticity and pathogenicity of the plant pathogenic bacterium *Xanthomonas campestris* pv. *vesicatoria* revealed by the complete genome sequence. J Bacteriol 187: 7254–7266. 16237009 10.1128/JB.187.21.7254-7266.2005 PMC1272972

[6] Potnis N, Krasileva K, Chow V, Almeida NF, Patil PB, et al.. (2011) Comparative genomics reveals diversity among xanthomonads infecting tomato and pepper. BMC Genomics: 146. 10.1186/1471-2164-12-146 PMC3071791 21396108

[7] Rodriguez LM, Koebnik R (2010) The *Xanthomonas* Resource. http://www.xanthomonas.org.

[8] Mudgett MB (2005) New insights to the function of phytopathogenic bacterial type III effectors in plants. Annu Rev Plant Biol 56: 509–531. 15862106 10.1146/annurev.arplant.56.032604.144218

[9] Block A, Li G, Fu ZQ, Alfano JR (2008) Phytopathogen type III effector weaponry and their plant targets. Curr Opin Plant Biol 11: 396–403. 18657470 10.1016/j.jbi.2008.06.007 PMC2570165

[10] Jones JD, Dangl JL (2006) The plant immune system. Nature 444: 323–329. 17108957 10.1038/nature05286

[11] Dodds PN, Rathjen JP (2010) Plant immunity: towards an integrated view of plant-pathogen interactions. Nat Rev Genet 11: 539–548. 20585331 10.1038/nrg2812

[12] Boller T, Felix G (2009) A renaissance of elicitors: perception of microbe-associated molecular patterns and danger signals by pattern-recognition receptors. Annu Rev Plant Biol 60: 379–406. 19400727 10.1146/annurev.arplant.57.032905.105346

[13] Essmann J, Schmitz-Thom I, Schon H, Sonnewald S, Weis E, et al (2008) RNA interference-mediated repression of cell wall invertase impairs defense in source leaves of tobacco. Plant Physiol 147: 1288–1299. 18502974 10.1104/pp.108.121418 PMC2442523

[14] Herbers K, Meuwly P, Frommer WB, Metraux JP, Sonnewald U (1996a) Systemic Acquired Resistance Mediated by the Ectopic Expression of Invertase: Possible Hexose Sensing in the Secretory Pathway. Plant Cell 8: 793–803. 12239401 10.1105/tpc.8.5.793 PMC161138

[15] Bolton MD (2009) Primary metabolism and plant defense–fuel for the fire. Mol Plant Microbe Interact 22: 487–497. 19348567 10.1094/MPMI-22-5-0487

[16] Horsfall JG, Dimond AE (1957) Interactions of tissue sugar, growth substances, and disease susceptibility. Zeitschrift für Pflanzenkrankheiten und Pflanzenschutz 64: 415–421.

[17] Sturm A, Tang GQ (1999) The sucrose-cleaving enzymes of plants are crucial for development, growth and carbon partitioning. Trends Plant Sci 4: 401–407. 10498964 10.1016/s1360-1385(99)01470-3

[18] Scharte J, Schon H, Weis E (2005) Photosynthesis and carbohydrate metabolism in tobacco leaves during an incompatible interaction with *Phytophthora nicotianae* . Plant, Cell & Environment 28: 1421–1435.

[19] Chou H-M, Bundock N, Rolfe SA, Scholes JD (2000) Infection of *Arabidopsis thaliana* leaves with *Albugo candida* (white blister rust) causes a reprogramming of host metabolism. Molecular Plant Pathology 1: 99–113. 20572957 10.1046/j.1364-3703.2000.00013.x

[20] Herbers K, Takahata Y, Melzer M, Mock HP, Hajirezaei M, et al (2000) Regulation of carbohydrate partitioning during the interaction of potato virus Y with tobacco. Molecular Plant Pathology 1: 51–59. 20572950 10.1046/j.1364-3703.2000.00007.x

[21] Kocal N, Sonnewald U, Sonnewald S (2008) Cell wall-bound invertase limits sucrose export and is involved in symptom development and inhibition of photosynthesis during compatible interaction between tomato and *Xanthomonas campestris* pv *vesicatoria* . Plant Physiol 148: 1523–1536. 18784281 10.1104/pp.108.127977 PMC2577280

[22] Swarbrick PJ, Schulze-Lefert P, Scholes JD (2006) Metabolic consequences of susceptibility and resistance (race-specific and broad-spectrum) in barley leaves challenged with powdery mildew. Plant, Cell & Environment 29: 1061–1076. 10.1111/j.1365-3040.2005.01472.x 17080933

[23] Roitsch T, Gonzalez MC (2004) Function and regulation of plant invertases: sweet sensations. Trends Plant Sci 9: 606–613. 15564128 10.1016/j.tplants.2004.10.009

[24] Sinha AK, Hofmann MG, Romer U, Kockenberger W, Elling L, et al (2002) Metabolizable and non-metabolizable sugars activate different signal transduction pathways in tomato. Plant Physiol 128: 1480–1489. 11950996 10.1104/pp.010771 PMC154275

[25] Ehness R, Ecker M, Godt DE, Roitsch T (1997) Glucose and Stress Independently Regulate Source and Sink Metabolism and Defense Mechanisms via Signal Transduction Pathways Involving Protein Phosphorylation. Plant Cell 9: 1825–1841. 12237349 10.1105/tpc.9.10.1825 PMC157025

[26] Godt DE, Roitsch T (1997) Regulation and tissue-specific distribution of mRNAs for three extracellular invertase isoenzymes of tomato suggests an important function in establishing and maintaining sink metabolism. Plant Physiol 115: 273–282. 9306701 10.1104/pp.115.1.273 PMC158483

[27] Roitsch T, Balibrea ME, Hofmann M, Proels R, Sinha AK (2003) Extracellular invertase: key metabolic enzyme and PR protein. J Exp Bot 54: 513–524. 12508062 10.1093/jxb/erg050

[28] Seo YS, Cho JI, Lee SK, Ryu HS, Han M, et al (2007) Current insight into primary carbon flux that occurs in plants undergoing a defense response. Plant Stress 1: 42–49.

[29] Rolland F, Baena-Gonzalez E, Sheen J (2006) Sugar sensing and signaling in plants: conserved and novel mechanisms. Annu Rev Plant Biol 57: 675–709. 16669778 10.1146/annurev.arplant.57.032905.105441

[30] Berger S, Sinha AK, Roitsch T (2007) Plant physiology meets phytopathology: plant primary metabolism and plant pathogen interactions. J Exp Bot 58: 4019–4026. 18182420 10.1093/jxb/erm298

[31] Biemelt S, Sonnewald U (2006) Plant-microbe interactions to probe regulation of plant carbon metabolism. J Plant Physiol 163: 307–318. 16368160 10.1016/j.jplph.2005.10.011

[32] Dean P (2012) Functional domains and motifs of bacterial type III effector proteins and their roles in infection. FEMS Microbiol Rev 35: 1100–1125. 10.1111/j.1574-6976.2011.00271.x 21517912

[33] Feng F, Zhou JM (2012) Plant-bacterial pathogen interactions mediated by type III effectors. Curr Opin Plant Biol 15: 469–476. 22465133 10.1016/j.pbi.2012.03.004

[34] Noel L, Thieme F, Gabler J, Buttner D, Bonas U (2003) XopC and XopJ, two novel type III effector proteins from *Xanthomonas campestris* pv. *vesicatoria* . J Bacteriol 185: 7092–7102. 14645268 10.1128/JB.185.24.7092-7102.2003 PMC296255

[35] Galan JE (2009) Common themes in the design and function of bacterial effectors. Cell Host Microbe 5: 571–579. 19527884 10.1016/j.chom.2009.04.008 PMC2729653

[36] Kay S, Bonas U (2009) How *Xanthomonas* type III effectors manipulate the host plant. Curr Opin Microbiol 12: 37–43. 19168386 10.1016/j.mib.2008.12.006

[37] Kay S, Hahn S, Marois E, Wieduwild R, Bonas U (2009) Detailed analysis of the DNA recognition motifs of the *Xanthomonas* type III effectors AvrBs3 and AvrBs3Deltarep16. Plant J 59: 859–871. 19473322 10.1111/j.1365-313X.2009.03922.x

[38] Kearney B, Staskawicz BJ (1990) Widespread distribution and fitness contribution of *Xanthomonas campestris* avirulence gene avrBs2. Nature 346: 385–386. 2374611 10.1038/346385a0

[39] Zhao B, Dahlbeck D, Krasileva KV, Fong RW, Staskawicz BJ (2011) Computational and biochemical analysis of the *Xanthomonas* effector AvrBs2 and its role in the modulation of *Xanthomonas* type three effector delivery. PLoS Pathog 7: e1002408. 22144898 10.1371/journal.ppat.1002408 PMC3228805

[40] Kay S, Hahn S, Marois E, Hause G, Bonas U (2007) A bacterial effector acts as a plant transcription factor and induces a cell size regulator. Science 318: 648–651. 17962565 10.1126/science.1144956

[41] Kim JG, Li X, Roden JA, Taylor KW, Aakre CD, et al (2009) *Xanthomonas* T3S Effector XopN Suppresses PAMP-Triggered Immunity and Interacts with a Tomato Atypical Receptor-Like Kinase and TFT1. Plant Cell 21: 1305–1323. 19366901 10.1105/tpc.108.063123 PMC2685636

[42] Taylor KW, Kim JG, Su XB, Aakre CD, Roden JA, et al (2012) Tomato TFT1 Is Required for PAMP-Triggered Immunity and Mutations that Prevent T3S Effector XopN from Binding to TFT1 Attenuate *Xanthomonas* Virulence. PLoS Pathog 8: e1002768. 22719257 10.1371/journal.ppat.1002768 PMC3375313

[43] Bartetzko V, Sonnewald S, Vogel F, Hartner K, Stadler R, et al (2009) The *Xanthomonas campestri*s pv. *vesicatoria* type III effector protein XopJ inhibits protein secretion: evidence for interference with cell wall-associated defense responses. Mol Plant Microbe Interact 22: 655–664. 19445590 10.1094/MPMI-22-6-0655

[44] Metz M, Dahlbeck D, Morales CQ, Al Sady B, Clark ET, et al (2005) The conserved *Xanthomonas campestris* pv. *vesicatoria* effector protein XopX is a virulence factor and suppresses host defense in *Nicotiana benthamiana* . Plant J 41: 801–814. 15743446 10.1111/j.1365-313X.2005.02338.x

[45] Salomon D, Dar D, Sreeramulu S, Sessa G (2011) Expression of *Xanthomonas campestris* pv. *vesicatoria* type III effectors in yeast affects cell growth and viability. Mol Plant Microbe Interact 24: 305–314. 21062109 10.1094/MPMI-09-10-0196

[46] Rossier O, Van den Ackerveken G, Bonas U (2000) HrpB2 and HrpF from *Xanthomonas* are type III-secreted proteins and essential for pathogenicity and recognition by the host plant. Mol Microbiol 38: 828–838. 11115117 10.1046/j.1365-2958.2000.02173.x

[47] Herbers K, Meuwly P, Metraux JP, Sonnewald U (1996b) Salicylic acid-independent induction of pathogenesis-related protein transcripts by sugars is dependent on leaf developmental stage. FEBS Lett 397: 239–244. 8955355 10.1016/s0014-5793(96)01183-0

[48] Nguyen HP, Chakravarthy S, Velasquez AC, McLane HL, Zeng L, et al (2010) Methods to study PAMP-triggered immunity using tomato and *Nicotiana benthamiana* . Mol Plant Microbe Interact 23: 991–999. 20615110 10.1094/MPMI-23-8-0991

[49] Kovach ME, Elzer PH, Hill DS, Robertson GT, Farris MA, et al (1995) Four new derivatives of the broad-host-range cloning vector pBBR1MCS, carrying different antibiotic-resistance cassettes. Gene 166: 175–176. 8529885 10.1016/0378-1119(95)00584-1

[50] Höfgen R, Willmitzer L (1990) Biochemical and genetic analysis of different patattin isoforms expressed in various organs of potato (*Solanum tuberosum*) Plant Sci. 66: 221–230.

[51] Caddick MX, Greenland AJ, Jepson I, Krause KP, Qu N, et al (1998) An ethanol inducible gene switch for plants used to manipulate carbon metabolism. Nat Biotechnol 16: 177–180. 9487526 10.1038/nbt0298-177

[52] Hofius D, Maier AT, Dietrich C, Jungkunz I, Bornke F, et al (2007) Capsid protein-mediated recruitment of host DnaJ-like proteins is required for Potato virus Y infection in tobacco plants. J Virol 81: 11870–11880. 17715215 10.1128/JVI.01525-07 PMC2168797

[53] Jakobek JL, Smith JA, Lindgren PB (1993) Suppression of Bean Defense Responses by *Pseudomonas syringae* . Plant Cell 5: 57–63. 12271016 10.1105/tpc.5.1.57 PMC160250

[54] Brown I, Mansfield J, Bonas U (1995) Hrp genes in *Xanthomonas campestris* pv *vesicatoria* determine ability to suppress papilla deposition in pepper mesophyll cells. Molecular Plant-Microbe Interactions 8: 825–836.

[55] Hauck P, Thilmony R, He SY (2003) A *Pseudomonas syringae* type III effector suppresses cell wall-based extracellular defense in susceptible Arabidopsis plants. Proc Natl Acad Sci U S A 100: 8577–8582. 12817082 10.1073/pnas.1431173100 PMC166271

[56] Bolouri Moghaddam MR, Van den Ende W (2012) Sugars and plant innate immunity. J Exp Bot. 63: 3989–3998. 10.1093/jxb/ers129 22553288

[57] Rausch T, Greiner S (2004) Plant protein inhibitors of invertases. Biochimica et Biophysica Acta-Proteins and Proteomics 1696: 253–261. 10.1016/j.bbapap.2003.09.017 14871666

[58] Huang LF, Bocock PN, Davis JM, Koch KE (2007) Regulation of invertase: a suite of transcriptional and post-transcriptional mechanisms. Functional Plant Biology 34: 499–507. 10.1071/FP06227 32689379

[59] Bonfig KB, Gabler A, Simon UK, Luschin-Ebengreuth N, Hatz M, Berger S, Muhammad N, Zeier J, Sinha AK, Roitsch T (2010) Post-translational derepression of invertase activity in source leaves via down-regulation of invertase inhibitor expression is part of the plant defense response. Mol Plant 3: 1037–1048. 20833735 10.1093/mp/ssq053

[60] Bonfig KB, Schreiber U, Gabler A, Roitsch T, Berger S (2006) Infection with virulent and avirulent *P. syringae* strains differentially affects photosynthesis and sink metabolism in Arabidopsis leaves. Planta 225: 1–12. 16807755 10.1007/s00425-006-0303-3

[61] Erbs G, Silipo A, Aslam S, De Castro C, Liparoti V, et al (2008) Peptidoglycan and muropeptides from pathogens *Agrobacterium* and *Xanthomonas* elicit plant innate immunity: structure and activity. Chem Biol 15: 438–448. 18482696 10.1016/j.chembiol.2008.03.017

[62] Keshavarzi M, Soylu S, Brown I, Bonas U, Nicole M, et al (2004) Basal defenses induced in pepper by lipopolysaccharides are suppressed by *Xanthomonas campestris* pv. *vesicatoria* . Mol Plant Microbe Interact 17: 805–815. 15242175 10.1094/MPMI.2004.17.7.805

[63] Sun W, Dunning FM, Pfund C, Weingarten R, Bent AF (2006) Within-species flagellin polymorphism in *Xanthomonas campestris* pv *campestris* and its impact on elicitation of Arabidopsis FLAGELLIN SENSING2-dependent defenses. Plant Cell 18: 764–779. 16461584 10.1105/tpc.105.037648 PMC1383648

[64] Felix G, Duran JD, Volko S, Boller T (1999) Plants have a sensitive perception system for the most conserved domain of bacterial flagellin. Plant J 18: 265–276. 10377992 10.1046/j.1365-313x.1999.00265.x

[65] Buttner D, Nennstiel D, Klusener B, Bonas U (2002) Functional analysis of HrpF, a putative type III translocon protein from *Xanthomonas campestris* pv. *vesicatori*a. J Bacteriol 184: 2389–2398. 11948151 10.1128/JB.184.9.2389-2398.2002 PMC135000

[66] Smeekens S, Ma J, Hanson J, Rolland F (2009) Sugar signals and molecular networks controlling plant growth. Curr Opin Plant Biol 13: 274–279. 10.1016/j.pbi.2009.12.002 20056477

[67] Thieme F, Szczesny R, Urban A, Kirchner O, Hause G, et al (2007) New type III effectors from *Xanthomonas campestris* pv. *vesicatoria* trigger plant reactions dependent on a conserved N-myristoylation motif. Mol Plant Microbe Interact 20: 1250–1261. 17918627 10.1094/MPMI-20-10-1250

[68] Kim JG, Taylor KW, Hotson A, Keegan M, Schmelz EA, et al (2008) XopD SUMO protease affects host transcription, promotes pathogen growth, and delays symptom development in xanthomonas-infected tomato leaves. Plant Cell 20: 1915–1929. 18664616 10.1105/tpc.108.058529 PMC2518228

[69] Hotson A, Chosed R, Shu H, Orth K, Mudgett MB (2003) *Xanthomonas* type III effector XopD targets SUMO-conjugated proteins in planta. Mol Microbiol 50: 377–389. 14617166 10.1046/j.1365-2958.2003.03730.x

[70] Noel L, Thieme F, Nennstiel D, Bonas U (2001) cDNA-AFLP analysis unravels a genome-wide hrpG-regulon in the plant pathogen *Xanthomonas campestris* pv. *vesicatoria* . Mol Microbiol 41: 1271–1281. 11580833 10.1046/j.1365-2958.2001.02567.x

[71] Schulze S, Kay S, Buttner D, Egler M, Eschen-Lippold L, et al (2012) Analysis of new type III effectors from *Xanthomonas* uncovers XopB and XopS as suppressors of plant immunity. New Phytol. 195: 894–911. 10.1111/j.1469-8137.2012.04210.x 22738163

[72] Nomura K, Debroy S, Lee YH, Pumplin N, Jones J, et al (2006) A bacterial virulence protein suppresses host innate immunity to cause plant disease. Science 313: 220–223. 16840699 10.1126/science.1129523

[73] Deblaere R, Bytebier B, DeGreve H, Beboeck F, Schell J, et al (1985) Efficient octopine Ti plasmid-derived vectors for Agrobacterium mediated gene transfer to plants. Nucleic Acids Res 13: 4777–4788. 4022773 10.1093/nar/13.13.4777 PMC321826

[74] Huguet E, Hahn K, Wengelnik K, Bonas U (1998) hpaA mutants of *Xanthomonas campestris* pv. *vesicatoria* are affected in pathogenicity but retain the ability to induce host-specific hypersensitive reaction. Mol Microbiol 29: 1379–1390. 9781876 10.1046/j.1365-2958.1998.01019.x

[75] Chen S, Hofius D, Sonnewald U, Bornke F (2003) Temporal and spatial control of gene silencing in transgenic plants by inducible expression of double-stranded RNA. Plant J 36: 731–740. 14617073 10.1046/j.1365-313x.2003.01914.x

[76] Rosahl S, Schell J, Willmitzer L (1987) Expression of a tuber-specific storage protein in transgenic tobacco plants: demonstration of an esterase activity. Embo J 6: 1155–1159. 16453760 10.1002/j.1460-2075.1987.tb02348.x PMC553913

[77] Logemann J, Schell J, Willmitzer L (1987) Improved method for the isolation of RNA from plant tissues. Anal Biochem 163: 16–20. 2441623 10.1016/0003-2697(87)90086-8

[78] Hajirezaei MR, Bornke F, Peisker M, Takahata Y, Lerchl J, et al (2003) Decreased sucrose content triggers starch breakdown and respiration in stored potato tubers (*Solanum tuberosum*). J Exp Bot 54: 477–488. 12508058 10.1093/jxb/erg040

[79] Ferreira SJ, Senning M, Sonnewald S, Kessling PM, Goldstein R, et al (2010) Comparative transcriptome analysis coupled to X-ray CT reveals sucrose supply and growth velocity as major determinants of potato tuber starch biosynthesis. BMC Genomics 11: 93. 20137087 10.1186/1471-2164-11-93 PMC2827413

[80] Mason G, Noris E, Lanteri S, Acquadro A, Accotto GP, et al (2008) Potentiality of Methylation-sensitive Amplification Polymorphism (MSAP) in Identifying Genes Involved in Tomato Response to Tomato Yellow Leaf Curl Sardinia Virus. Plant Molecular Biology Reporter 26: 156–173.

[81] Untergasser A, Nijveen H, Rao X, Bisseling T, Geurts R, et al (2007) Primer3Plus, an enhanced web interface to Primer3. Nucleic Acids Res 35: W71–74. 17485472 10.1093/nar/gkm306 PMC1933133

[82] Canteros B (1990) Diversity of plasmids and plasmid-encoded traits in *Xanthomonas campestris* pv. *vesicatoria*. Grainsville, FL, USA: University of Florida.

[83] Roden JA, Belt B, Ross JB, Tachibana T, Vargas J, et al (2004) A genetic screen to isolate type III effectors translocated into pepper cells during *Xanthomonas* infection. Proc Natl Acad Sci U S A 101: 16624–16629. 15545602 10.1073/pnas.0407383101 PMC534543

