# Supplementary figures and images for: Regulation of Cell Wall-Bound Invertase in Pepper Leaves by Xanthomonas campestris pv. vesicatoria Type Three Effectors
Source: PLoS One. 2012 Dec 14;7(12):e51763. doi: 10.1371/journal.pone.0051763 (PMC3522709; doi:10.1371/journal.pone.0051763)

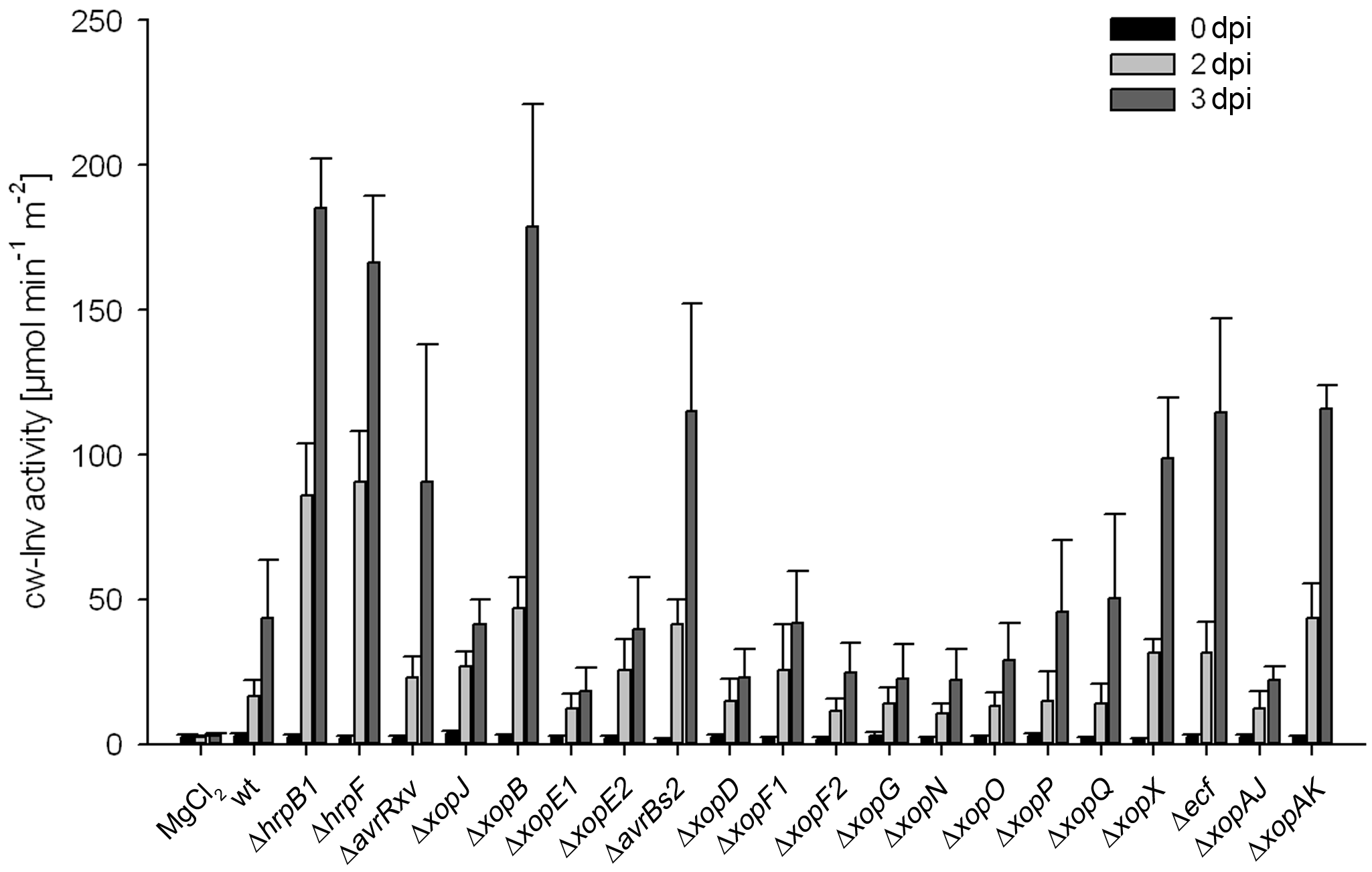

Supplement: Figure S1 — Cw-Inv activity in pepper leaves after infiltration with Xcv wild type and Xcv mutant strains at a concentration of 109 cfu ml−1. Values represent the mean ± SD from two different experiments each with four independent samples. Black bars: before infection. Light grey: 2 days post infection (dpi). Dark grey: 3 dpi. (TIF) [file pone.0051763.s001.tif]

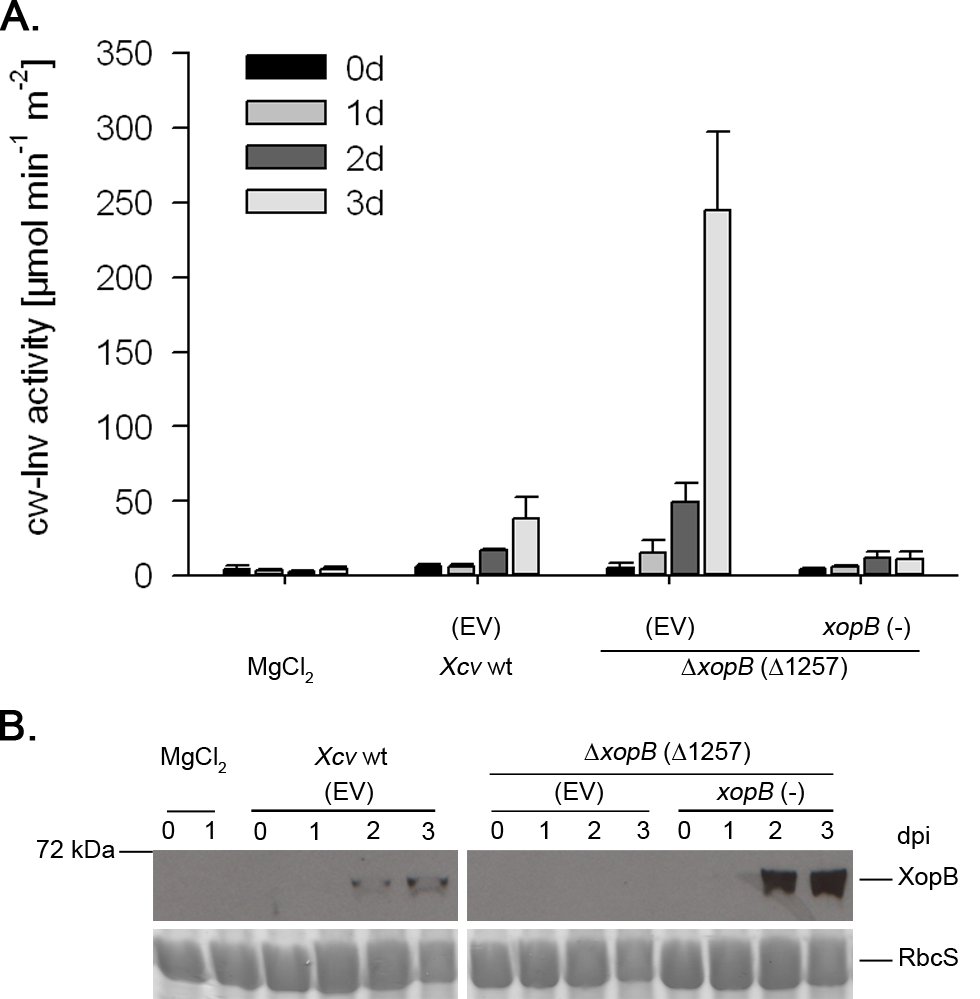

Supplement: Figure S2 — Xcv Δ xopB (Δ1257) induces a strong induction of cw-Inv activity which is complemented by plasmid-borne expression of XopB. Susceptible pepper leaves were inoculated with Xcv wild type (wt), Xcv ΔxopB (Δ1257) containing both the pBBR1MCS5 vector (EV), or a Xcv ΔxopB (Δ1257) derivative in which a genomic fragment was introduced containing the putative xopB promoter and open reading frame in antisense (−) orientation relative to the lac promoter. Samples were taken before (0) and 1, 2, 3 days post infection (dpi). A.) Cw-Inv activity was measured from the infected leaf tissue. Values represent the mean of four independent samples ± SD. B.) Expression of XopB was verified by Western blotting by probing with the anti-XopB antibody. XopB migrates at ∼70 kDa. (TIF) [file pone.0051763.s002.tif]

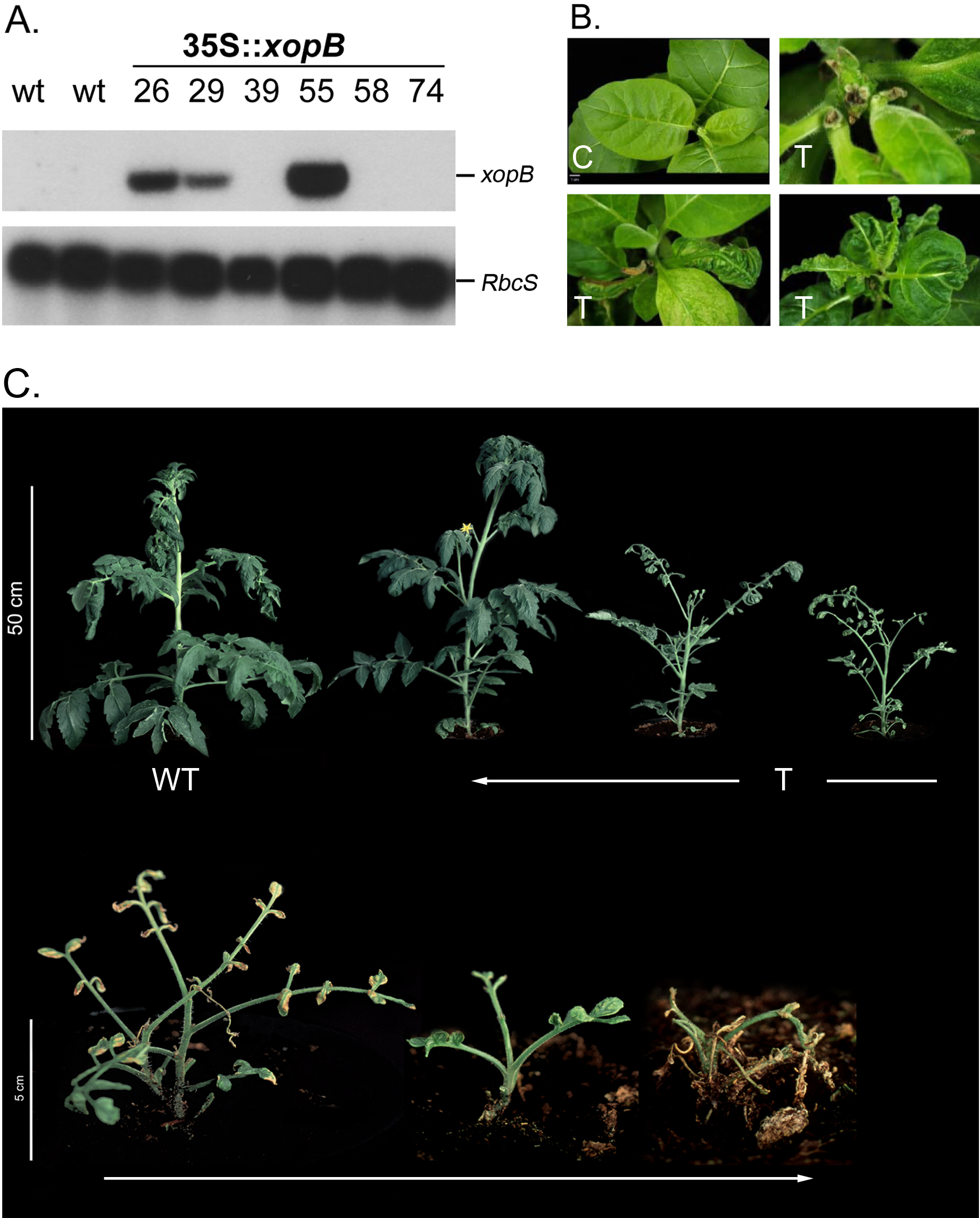

Supplement: Figure S3 — Constitutive expression of xopB in transgenic tobacco and tomato plants. The xopB open reading frame was inserted into the binary vector pBinAR between the CaMV 35S promoter and the polyadenylation site of the octopin synthase from Agrobacterium tumefaciens and transformed into tobacco and tomato plants. A.) Analysis of xopB-specific mRNA accumulation in transgenic tobacco lines. Total RNA was isolated from six different transgenic lines and from two wild type plants (wt). Expression of xopB using a gene-specific probe was verified by Northern blotting. B.) Phenotypic changes in transgenic tobacco plants caused by constitutive xopB mRNA expression. Shown are three independent lines compared to wild type (upper left panel). C.) Phenotypic changes in transgenic tomato plants caused by constitutive xopB expression. Shown are wild type (upper left panel) and several transgenic lines with different level of xopB expression. (TIF) [file pone.0051763.s003.tif]
