# Supplementary material for: Regulation of Cell Wall-Bound Invertase in Pepper Leaves by Xanthomonas campestris pv. vesicatoria Type Three Effectors
Source: PLoS One. 2012 Dec 14;7(12):e51763. doi: 10.1371/journal.pone.0051763 (PMC3522709; doi:10.1371/journal.pone.0051763)
Supplement: Table S1 — Primers used for generation of different Xcv strains. Added restriction sites were marked by bold letters. (DOCX) [file pone.0051763.s004.docx]

**Table S1**. Primers used for generation of different *Xcv* strains.

Added restriction sites were marked in bold letters.

| Strain | Gene ID | Characteristics | Primer Sequences |
| --- | --- | --- | --- |
| *Xcv* Δ*avrBs2* | XCV0052 | 1554bp deletion (start: bp 228;end: bp 1782) | 5’P1_avrBs2 (BamHI) **GGA TCC** ATG CGT ATC GGT CCT CTG |
|  |  |  | 3’P1_avrBs2GGC CTC GGG CTG ATC GGT GGA GAA TTC GCT GTC GAG |
|  |  |  | 5’P2_AvrBs2CTC GAC AGC GAA TTC TCC ACC GAT CAG CCC GAG GCC |
|  |  |  | 3’P2_avrBs2 (SalI) **GTC GAC** TCA ATC CGT CTC CGT CTG |
| *Xcv* Δ*avrRxv* | XCV0471 | 241bp deletion (start: bp 539;end: bp 780) | 5’P1_avr (BamHI) **GGATCC**ATGTGCGACTCCATAAGAGTG |
|  |  |  | 3’P1_avr CCATTGGTCAAAGGTATGCCATCTCCTAGGGTC |
|  |  |  | 5’P2_avr GACCCTAGGAGATGGCATACCTTTGACCAATGG |
|  |  |  | 3’P2_avr (SalI/ XbaI) **TCGACTCTAGA**TCAGGATTCTAAGGCGTGACGGAT |
| *Xcv* Δ*xopE1* | XCV0294 | 786bp deletion (start: bp 195;end: bp 981) | 5’P1_xopE1 (BamHI) **GGA TCC** ATG GGA CTA TGC ATT TC |
|  |  |  | 3’P1_xopE1 TCC AGT CCG TAG GCT TGG GTC GGT TTC CCG GAG C |
|  |  |  | 5’P2_xopE1 GCT CCG GGA AAC CGA CCC AAG CCT ACG GAC TGG A |
|  |  |  | 3’P2_xopE1 (XbaI) **TCT AGA** TCA TCT CGC CAC CGT GAC |
| *Xcv* Δ*xopE2* | XCV2280 | 445bp deletion (start: bp 347; end: bp 792) | 5’P1_xopE2 (BamHI) **GGA TCC** ATG GGG CTA TGC AGT TC |
|  |  |  | 3’P1_xopE2 GCA TTC GCA TGG AGA TCC CTC GTC CAC GGT GCA A |
|  |  |  | 5’P2_xopE2 TTG CAC CGT GGA CGA GGG ATC TCC ATG CGA ATG C |
|  |  |  | 3’P2_xopE2 (XbaI) **TCT AGA** TCA CCA TCT CAA GGG TGG |
| *Xcv* Δ*xopF2* | XCV2942 | 1380bp deletion (start: bp 231; end: bp 1611) | 5’P1_xopF2 (BamHI) **GGA TCC** ATG AAG CTC CAA CGC CAG |
|  |  |  | 3’P1_xopF2 CTC GTG ATC CAA TCG CGT CGG ACG CCA CTG CCT GGT |
|  |  |  | 5’P2_xopF2 ACC AGG CAG TGG CGT CCG ACG CGA TTG GAT CAC GAG |
|  |  |  | 3’P2_xopF2 (BamHI) **GGA TCC** TCA AGG CCT ACC CTG TTG |
| *Xcv* Δ*xopG* | XCV1298 | 297bp deletion (start: bp 138; end: bp 546) | 5’P1_xopG (BamHI) **GGA TCC** CGA CAT GTC AGC CTC CTC |
|  |  |  | 3’P1_xopG GGT CTT TCG ATA TTC GTA TTC TAG AAG GGC ACT GCC |
|  |  |  | 5’P2_xopG GGC AGT GCC CTT CTA GAA TAC GAA TAT CGA AAG ACC |
|  |  |  | 3’P2_xopG (SalI) **GTC GAC** GCA TGC TGG GCA CTT GTG |
| *Xcv* Δ*xopX* | XCV0572 | 1290bp deletion (start: bp 351; end: bp 1641) | 5’P1_xopX (BamHI) **GGA TCC** ATG GAG ATC AAG AAA CAG |
|  |  |  | 3’P1_xopX GGC GTA GTA GGG CGA TGA CGA CCA GAG ATT GGA CGC |
|  |  |  | 5’P2_xopX GCG TCC AAT CTC TGG TCG TCA TCG CCC TAC TAC GCC |
|  |  |  | 3’P2_xopX (SalI) **GTC GAC** TCA GGA CGA AGG CAC AGT |
| *Xcv* Δ*ecf* | XCV3785 | 1227bp deletion (start: bp 363; end 1590 bp) | 5’P1_ecf (XbaII) G **TCT AGA** ATG CAG ATC AAA ACC GCA G |
|  |  |  | 3’P1_ecf GTC GTC CAT GTA GCC GCT CGA GCA ATC ACG CAT GAG |
|  |  |  | 5’P2_ecf CTC ATG CGT GAT TGC TCG AGC GGC TAC ATG GAC GAC |
|  |  |  | 3’P2_ecf (BamHI) **GGA TCC** TTA TTC CGA CTG AGG CAC |
| *Xcv* Δ*xopAJ (avrRxoI)* | XCV4428 | 651bp deletion (start: bp 411; end: bp 1062) | 5’P1_avrRxoI-n (BamHI) **GGA TCC** TTC GTG GGT GTG CGA GTC |
|  |  |  | 3’P1_avrRxoI_n GAA TAG TCG ATA GTT TCC CGG ACC AGA AGA TGG GAG |
|  |  |  | 5’P2_avrRxoI-n CTC CCA TCT TCT GGT CCG GGA AAC TAT CGA CTA TTC |
|  |  |  | 3’P2_avrRxoI_alt (SalI) **GTC GAC** TCA AAT TAG CTC GCT ATC |
| *Xcv* Δ*xopAK* | XCV3786 | 690bp deletion (start: bp 354; end: bp 1044) | 5’P1_Xcv3786 (BamHI) **GGA TCC** ATG TGC GTT GCC AGG CCT C |
|  |  |  | 3’P1_Xcv3786 GAT GAC GAC ACA TGC GTC CGC TCG ATC GTT CCC GAT |
|  |  |  | 5’P2_Xcv3786 ATC GGG AAC GAT CGA GCG GAC GCA TGT GTC GTC ATC |
|  |  |  | 3’P2_Xcv3786 (SalI) **GTC GAC** TCA CCA CGA CTT GTA GTA |
| *Xcv* Δ*xopB* (Δ1257) | XCV0581 | 1257bp deletion (start: bp 259; end: bp 1515) | XopB_5’ P1 (BamHI) **GGA TCC** atg aag gca gag ctc aca c |
|  |  |  | xopB_3’ P1 GAC ATG AAT GCG TCC TAG CGG AGT TTC TAA CCC |
|  |  |  | xopB_5’ P2 ggg tta gaa act ccg cta gga cgc att cat gtc |
|  |  |  | xopB_3’ P2 (SalI) **GTC GAC** TTA CGG CTC AGG CGC GGG |
| pBBR::xopB(+) (sense); pBBR::xopB(-) (antisense) |  | 2598 bp genomic fragment Start: -649bp End: 1949bp | xopB_compl_5’_BamHI GC **GGA TCC** TGA AGT ACG CTG GCC TTG CGC xopB_compl_3’_BamHI GC **GGA TCC** TCG ACC GCA GAC CCC TAA TTC |
